# CD36 restricts lipid-associated macrophages accumulation in white adipose tissues during atherogenesis

**DOI:** 10.3389/fcvm.2024.1436865

**Published:** 2024-08-02

**Authors:** Vaya Chen, Jue Zhang, Jackie Chang, Mirza Ahmar Beg, Lance Vick, Dandan Wang, Ankan Gupta, Yaxin Wang, Ziyu Zhang, Wen Dai, Mindy Kim, Shan Song, Duane Pereira, Ze Zheng, Komal Sodhi, Joseph I. Shapiro, Roy L. Silverstein, Subramaniam Malarkannan, Yiliang Chen

**Affiliations:** ^1^ Versiti Blood Research Institute, Milwaukee, WI, United States; ^2^ Department of Biochemistry, Medical College of Wisconsin, Milwaukee, WI, United States; ^3^ Department of Microbiology and Immunology, Medical College of Wisconsin, Milwaukee, WI, United States; ^4^ Department of Pediatrics, Medical College of Wisconsin, Milwaukee, WI, United States; ^5^ Department of Physiology, Medical College of Wisconsin, Milwaukee, WI, United States; ^6^ Department of Pathology, Hebei Medical University, Shijiazhuang, China; ^7^ Hebei Key Laboratory of Kidney Diseases, Shijiazhuang, China; ^8^ Department of Surgery, Biomedical Sciences, and Medicine, Joan C. Edwards School of Medicine, Marshall University, Huntington, WV, United States; ^9^ Department of Medicine, Medical College of Wisconsin, Milwaukee, WI, United States

**Keywords:** lipid, inflammation, visceral adipose tissue, macrophage, scRNA-Seq, atherosclerosis

## Abstract

Visceral white adipose tissues (WAT) regulate systemic lipid metabolism and inflammation. Dysfunctional WAT drive chronic inflammation and facilitate atherosclerosis. Adipose tissue-associated macrophages (ATM) are the predominant immune cells in WAT, but their heterogeneity and phenotypes are poorly defined during atherogenesis. The scavenger receptor CD36 mediates ATM crosstalk with other adipose tissue cells, driving chronic inflammation. Here, we combined the single-cell RNA sequencing technique with cell metabolic and functional assays on major WAT ATM subpopulations using a diet-induced atherosclerosis mouse model (*Apoe*-null). We also examined the role of CD36 using *Apoe*/*Cd36* double-null mice. Based on transcriptomics data and differential gene expression analysis, we identified a previously undefined group of ATM displaying low viability and high lipid metabolism and labeled them as “unhealthy macrophages”. Their phenotypes suggest a subpopulation of ATM under lipid stress. We also identified lipid-associated macrophages (LAM), which were previously described in obesity. Interestingly, LAM increased 8.4-fold in *Apoe*/*Cd36* double-null mice on an atherogenic diet, but not in *Apoe*-null mice. The increase in LAM was accompanied by more ATM lipid uptake, reduced adipocyte hypertrophy, and less inflammation. In conclusion, CD36 mediates a delicate balance between lipid metabolism and inflammation in visceral adipose tissues. Under atherogenic conditions, CD36 deficiency reduces inflammation and increases lipid metabolism in WAT by promoting LAM accumulation.

## Introduction

Atherosclerosis is characterized by hyperlipidemia, oxidative stress, and chronic inflammation. It is the underlying mechanism causing a variety of cardiovascular diseases, which remain the leading cause of death worldwide (1). Although atherosclerotic plaques mainly develop in the medium and large arteries, many studies have shown that adipose tissues, especially the visceral white adipose tissues (WAT), contribute to atherosclerosis, possibly through their adverse effects on systemic lipid metabolism and immune regulation (2–4). Despite decades of studies, molecular mechanisms linking WAT phenotypes and functions to atherosclerosis development remain poorly understood.

Adipose tissues normally consist of adipocytes, which are the specialized lipid-storage cells, along with preadipocytes, vascular endothelial cells, fibroblasts, and many immune cells such as macrophages, dendritic cells, and lymphocytes (5). Among adipose tissue-associated immune cells, macrophages are the predominant cells, representing about 5% of all adipose tissue cells from lean mice and humans (6). Interestingly, the adipose tissue-associated macrophages (ATM) can undergo pro-inflammatory phenotypic switch (7) and expand dramatically up to over 50% of all adipose tissue cells in obesity (8), a significant risk factor for atherosclerosis (9, 10). New technologies such as single-cell RNA sequencing (scRNA-seq) have significantly advanced our knowledge of the dynamics of ATM in obesity. As an example, a recent report using obese mice identified a distinct ATM subpopulation called lipid-associated macrophages (LAM), which are active in lipid metabolism, and play a protective role against adipocyte hypertrophy and systemic inflammation (11). Unfortunately, while ATM pro-inflammatory activation and expansion under atherogenic conditions are well documented (12), the phenotypes and heterogeneity of ATM during atherogenesis remain poorly understood.

CD36 is a type 2 cell surface scavenger receptor highly expressed in macrophages as well as non-immune cells like adipocytes (13). Besides its role as a signal transducer mediating immune cell activation, CD36 also facilitates long-chain fatty acid transport across the plasma membrane, which is a unique feature among the scavenger receptors (14). We and our colleagues have previously demonstrated a contributing role of CD36 in foam cell formation and the development of atherosclerosis (15–18). Specifically, mice deficient in CD36 are resistant to diet-induced atherosclerosis (17). Moreover, CD36 deficiency protects mice ATM from diet-induced pro-inflammatory activation, implicating CD36 in the crosstalk between adipocytes and ATM (19).

In this work, we used scRNA-seq to analyze the transcriptomics of ATM from WAT of *Apoe*-null mice fed with an atherogenic high-fat diet (HFD), a widely used diet-induced atherosclerosis animal model (20). We identified the major subpopulations of ATM based on the specific transcriptome signatures described by others, including LAM, inflammatory macrophages, cavity macrophages, and vascular-associated macrophages (VAM). We also reported the presence of “unhealthy macrophages” displaying high lipid metabolism and low viability. Consistent with the literature on LAM in obesity (11), our pathway analysis revealed that the LAM were highly active in lipid metabolism, but downregulated pro-inflammatory pathways, compared to other major ATM. We further analyzed and identified the transcription factor regulons specific to each ATM subpopulation. Using ATM from WAT of age-matched *Apoe*/*Cd36* double-null mice (17), we found that HFD resulted in an 8.4-fold increase in the relative amount of LAM in *Apoe*/*Cd36* double-null mice, but not in *Apoe*-null mice. We validated this finding at protein levels by immunostaining and flow cytometry. Additionally, LAM increase was accompanied by a significant 42% higher long-chain fatty acid (palmitate) uptake by the ATM, less inflammatory ATM induction, and less adipocyte hypertrophy. Finally, we showed that the most differentially regulated genes caused by CD36 deficiency were those involved in pro-inflammatory responses and lipid metabolism. Our findings have indicated a negative role of CD36 in the regulation of LAM amount and lipid handling during atherogenesis and provided novel insights into molecular mechanisms of ATM dynamics.

## Materials and methods

### Mice

All mice used in this study were on the C57BL/6 background. *Apoe*-null mice (strain# 002052) were purchased from the Jackson Laboratory. *Apoe*/*Cd36* double-null mice were generated as previously described (17). All mice were kept in a 12-hour dark/light cycle and fed standard chow *ad libitum* until the start of the diet challenge. Adult mice of both sexes between 12 and 16 weeks of age were randomly divided into two groups: one was continued on a standard chow diet, and the other was placed on an HFD (Harlan Teklad, #TD.88137) for 10 weeks. The diet is high in total fat (21% by weight and 42% kcal from fat) and saturated fatty acids (>60% of total fatty acids) and is widely used on atherosclerosis-prone mouse models (16, 18). All mice were weighed once every week before being euthanized at the end of the 10-week diet challenge. Mice were euthanized by CO_2_ asphyxiation followed by cervical dislocation. For the scRNA-seq, we combined perigonadal visceral white adipose tissue WAT (pgWAT) from one male and one female in each condition to eliminate differences introduced by sex. All procedures involving live animals were approved by the Institutional Animal Care and Use Committee at the Medical College of Wisconsin.

### Isolation of pgWAT-associated leukocytes for scRNA-Seq

Mice were sacrificed and perfused with 10 ml PBS to remove peripheral blood from the adipose tissues. Then pgWAT was isolated, weighed, and washed with ice-cold PBS. pgWAT was minced into 2–3 mm pieces by scissors and transferred to gentleMACS C-tubes (Cat# 130-093-237, Miltenyi Biotec) with 1.5 ml enzymatic digestion mixture (Liberase, 0.77 mg/ml; Hyaluronidase, 0.3 mg/ml; Deoxyribonuclease, 0.3 mg/ml; BSA, 1 mg/ml; CaCl2, 1.5 μM in PBS). Tissues were digested in a gentle MACSTM Octo Dissociator with Heaters (Cat# 130-096-427, Miltenyi Biotec) at 37°C for 30 min. Suspensions were immediately filtered with a 70 μm cell strainer (Fisher Scientific) and washed once with FACS buffer (5% FBS and 0.1% sodium azide in PBS). Then, CD45^+^ cells were selected by the EasySep Mouse CD45 Positive Selection Kit (Cat# 18945, STEMCELL Technologies). The CD45^+^ cells were then suspended in the FACS buffer, counted, and immediately loaded onto a 10× Genomics Chromium instrument according to the instructions from the Chromium Next GEM Single Cell 3’ Reagent Kits v3.1 (Cat# PN-1000121, 10× Genomics, Pleasanton, CA, USA). 5,000 live cells per condition were loaded onto each GEM.

### Flow cytometry assays

Cell suspension was prepared in 200 μl FACS buffer. All flow cytometry experiments were performed with a BD LSR II instrument using FACSDiva software with optimal compensation and gain settings determined for each experiment based on unstained and single color-stained samples. Live cells were gated based on cell forward and side scatter signals. Doublets were excluded based on FSC-A vs. FSC-H plots. Flowjo software version 10.9.0 (Tree Star, OR) was used to analyze the data. To quantify Trem2^+^ LAM, cell suspension was stained with FITC-conjugated anti-F4/80 (Cat# 123108, Biolegend) and PE-conjugated anti-Trem2 (Cat# FAB17291P, R&D Systems). Cells were washed three times with FACS buffer and subjected to flow cytometry analysis.

### *Ex vivo* palmitate uptake assay

CD45 + cells isolated from pgWAT were incubated with 1 μg/ml Bodipy-C16 Palmitate (Cat# D3821, Invitrogen) in FACS buffer at 37°C 15 min (18) and stained by Pacific Blue-conjugated anti-CD11b (Cat# 101224, Biolegend) and PE/Cyanine5-conjugated anti-F4/80 (Cat# 123111, Biolegend). Cells were washed three times with FACS buffer and subjected to flow cytometry analysis.

### Plasma isolation and profiling

All mice were fast for 5 h before whole blood collection through the jugular vein. The whole blood was centrifuged at room temperature at 1,200 g for 10 min. The supernatant (plasma) was transferred to a fresh Eppendorf tube and stored at −80 freezer. Plasma insulin was measured using a commercial ELISA kit (Cat# 90080, Crystal Chem). Plasma IL-6 was measured by a commercial ELISA kit (Cat# 88-7064-22, Invitrogen).

### Single-cell RNA-sequencing analyses

#### Library preparation and sequencing

ScRNA-seq libraries were prepared using the Chromium Next GEM Single Cell 3’ Reagent Kits v3.1 (10× Genomics) following the manufacturer's instructions. The library quality was assessed with the Bioanalyzer RNA Nano Assay (Agilent). Generated libraries were then sequenced on the Illumina High Seq-2500 platform.

#### Data processing

Feature-barcode matrix were generated using the Cell Ranger 4.0 (10× Genomics). Sequenced data were aggregated and reads were aligned by the Cell Ranger. The following quality control steps were conducted: 1. cells that have unique feature counts over 6,000 or less than 50 were excluded for further analysis; 2. cells that have over 20% of unique molecular identifiers (UMIs) were derived from the mitochondrial genome were excluded for further analysis; 3. cells with the expression more than 4 in Hbb-related genes like Hbb-bs, Hba-a1, Hba-a2, and Hbb-bt after normalization were considered contaminated cells from epididymis and were excluded for further analysis. The data were normalized and scaled for principal component analysis (PCA) by Seurat Package. The computational method SingleR was used to annotate cell clusters. Then identified macrophages from all 4 conditions were pooled together for further analysis. We used 10 dims from PCA and set the resolution to 0.5 for cell clustering. Uniform manifold approximation and projection (UMAP) functions were used for visualizing the cells on a 2-dimension plot.

#### Gene set analysis and regulon analysis

We used gseKEGG function in ClusterProfile Package (21) for gene set enrichment analysis (GSEA), based on FindMarkers function from Seurat Package. Library org.Mm.eg.db was used for gene ID mapping. We used dotplot and gseaplot2 for further visualizing the GSEA results. Transcription factor regulons were characterized by Single-Cell rEgulatory Network Inference and Clustering (SCENIC), a computational method generated previously (22).

## Results

### Macrophages are the predominant immune cells in the white adipose tissues during atherogenesis

We fed *Apoe*-null mice with HFD for 10 weeks, a widely used diet-induced atherosclerosis animal model (20). CD36 is widely expressed in many immune cells, especially macrophages (13), and has a significant impact on adipose tissue inflammation and metabolism (19). Moreover, *Apoe*/*Cd36* double-null mice were resistant to diet-induced atherosclerosis (17). Therefore, in this work, we included *Apoe*/*Cd36* double-null mice and compare them with *Apoe*-null mice to investigate how CD36 deficiency affected ATM phenotypes. We chose the pgWAT for our scRNA-seq analysis because visceral WAT, compared with subcutaneous adipose tissues, are more correlated with metabolic syndromes and atherosclerosis (2, 3, 23). After a 10-week diet to induce an intermediate stage of atherosclerosis in the *Apoe*-null HFD group, we removed pgWAT from all 4 groups of mice (Supplementary Figure S1), digested to single cell suspension, and purified adipose tissue immune cells using a CD45 positive selection kit (StemCell Technologies). Immediately, we performed scRNA-seq using the 10× Genomics platform (Supplementary Figure S1). After the data quality control steps, we have altogether acquired 1,650 cells from *Apoe*-null chow pgWAT, 3,957 cells from *Apoe*-null HFD pgWAT, 1,759 cells from *Apoe*/*Cd36* double-null chow pgWAT, and 1,987 cells from *Apoe*/*Cd36* double-null HFD pgWAT.

**Figure 1:**
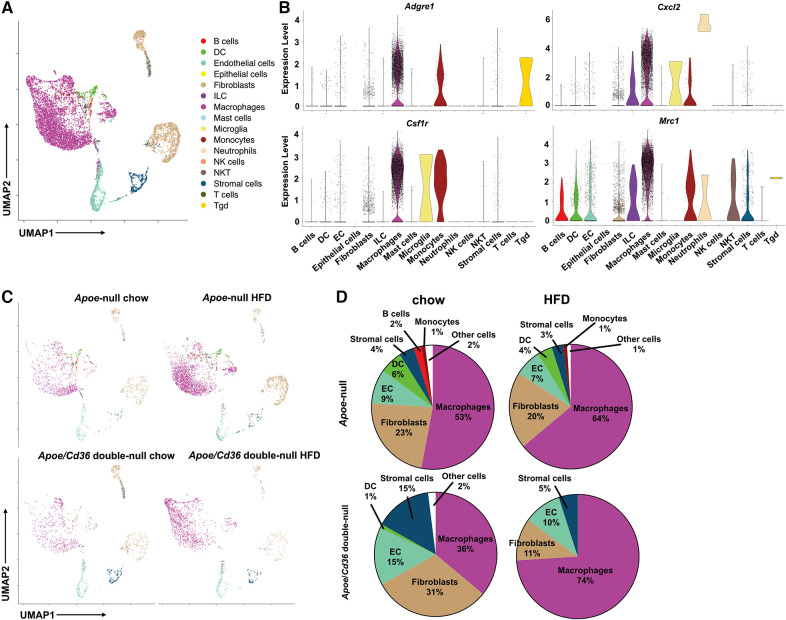
ATM represent the major immune cell type in the white adipose tissue during atherogenesis. (**A**) The *Apoe*-null or *Apoe*/*Cd36* double-null mice fed on chow diet or HFD for 10 weeks were sacrificed (*n* = 2 per group) and CD45^+^ cells were isolated from pgWAT and subjected to scRNA-Seq analysis. Cells were unbiasedly clustered based on the FindNeighbors method from Seurat package. Distinct cell clusters are shown through uniform manifold approximation and projection (UMAP). Immune cell types were determined using the SingleR method. (**B**) Violin plots of relative expression of macrophage marker genes among CD45^+^ adipose tissue cells. (**C**) UMAP plots of 4 different conditions were shown. (**D**) Percentages of immune cell populations as defined by SingleR within total CD45^+^ cells in each condition were shown.

Next, we identified each cell cluster using a previously developed computational method, SingleR (24), according to ImmGen reference database (25). We found that pgWAT contained a variety of immune cells, including B cells, T cells, macrophages, dendritic cells, innate lymphoid cells, neutrophils, NK cells, NKT cells, and other less-characterized cells. We used Seurat package to show distinct immune cell subpopulations through uniform manifold approximation and projection (UMAP) (Figure 1A). Violin plots of four commonly used macrophage markers (*Adgre1*, *Csf1r*, *Cxcl2*, and *Mrc1*) validated the macrophage identity (Figure 1B). Consistent with the literature (26), among all CD45^+^ pgWAT immune cells, macrophages represented the largest population (Figure 1C). Interestingly, macrophage relative amount was lower in *Apoe*/*Cd36* double-null chow (36%) compared to *Apoe*-null chow (53%), suggesting that CD36 expression was involved in macrophage differentiation or maintenance. However, the macrophage relative amount increased to 74% in *Apoe*/*Cd36* double-null HFD as compared to 64% in *Apoe*-null HFD (Figure 1D). Thus, CD36 appears to play a negative role in macrophage population expansion induced by HFD.

### Identification of pgWAT macrophage subpopulations

**Figure 2:**
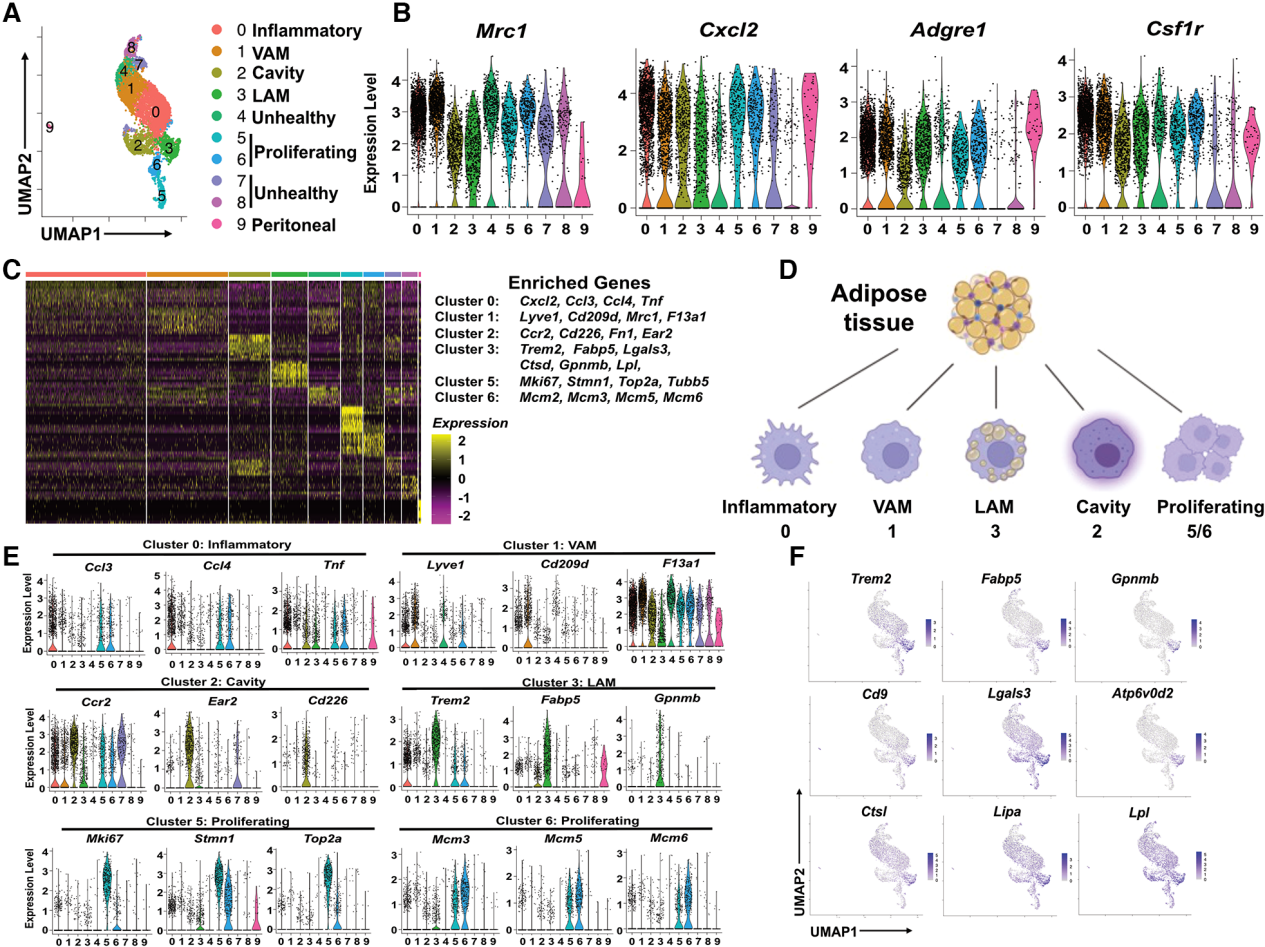
Identification of subpopulations of pgWAT ATM, which are highly dynamic during atherogenesis. (**A**) ATM from all 4 conditions were selected and combined. They were divided into 10 clusters (0–9) using the same method as in Figure 1 and shown in the UMAP. (**B**) Violin plots of relative expression of macrophage marker genes among ATM clusters. (**C**) Heatmap showing the 10 most upregulated genes in each cluster as defined in (**A**) Selected enriched genes used for biological identification of each cluster are shown on the right of the heatmap. (**D**) The ATM names corresponding to different cluster numbers in the texts are shown. (**E**) Violin plots of relative expression of enriched genes in the cluster 0, 1, 2, 3, 5, and 6. (**F**) Gene expression patterns projected onto the UMAP plots showing the enrichment of *Trem2*, *Fabp5*, *Gpnmb*, *Cd9*, *Lagls3*, *Atp6v0d2*, *Ctsl*, *Lipa*, and *Lpl* in LAM (cluster 3). The graphical panel in Figure 2D was created with BioRender.com.

Since ATM were the predominant immune cells in pgWAT and expanded further by HFD, we propose that change in macrophage subpopulations could provide critical knowledge on the inflammatory and metabolic status of pgWAT. To better characterize and identify macrophage subpopulations, we selected macrophages from all 4 samples and generated a second UMAP (Figure 2A). We detected 10 distinct macrophage clusters (Cluster 0–9). Among the 10 clusters, cluster 9 represented a very minor subset (<1%) with highly distinct transcriptomics from other clusters. Besides, their enriched genes showed a signature of peritoneal macrophages, i.e., *Gata6, Fn1, F5, Selp* (Supplementary Figure S2A), as reported previously (27, 28). Thus, we reasoned that cluster 9 represented peritoneal macrophage contamination during adipose tissue dissection and excluded cluster 9 from the following analysis. Clusters 4, 7, and 8 displayed relatively low feature counts and total RNA counts compared to other clusters (Supplementary Figure S2B), an indication of high cell membrane permeability or low cell viability. Despite that, these clusters (especially cluster 4) still expressed common macrophage markers such as *Mrc1, cxcl2, adgre1*, and *csf1r*, like other clusters (Figure 2B). So, we labeled clusters 4/7/8 as unhealthy macrophages. To explore the potential mechanism leading to the “unhealthiness”, we generated a volcano plot comparing the cluster 4/7/8 and other “healthy” ATM (Supplementary Figure S2C). In “unhealthy” macrophages, upregulated genes included those related to fatty acid transport (*Fabp4*), fatty acid synthesis (*Scd1*), and biogenesis of membrane lipid domains (*cav1*, *cavin1*, *cavin2*), which also affect lipid metabolism (29, 30). Downregulated genes included *Lgals3*, which encodes a protein with anti-apoptotic functions (31), and *Ftl1*, which encodes the ferritin light chain to protect cells from iron-induced oxidative stress and ferroptosis (32). Because ferroptosis is triggered by lipid peroxidation, the data implies that the unhealthy macrophages might be ferroptosis-prone cells due to abnormal lipid and iron metabolism. Next, we focused on the major clusters 0, 1, 2, 3, 5, and 6.

We generated a heatmap of the top 10 enriched genes for each cluster. The major marker genes for clusters 0–3, 5, and 6 were listed on the right side of the heatmap (Figure 2C) and were used to identify ATM subpopulations (Supplementary Table 1 and Figure 2D). Cluster 0 was enriched with pro-inflammatory chemokines *Ccl3*, *Ccl4*, Cxcl2, and pro-inflammatory cytokine *Tnf* (Figures 2C,E). Their enriched gene signature was similar to that of inflammatory macrophages associated with mouse aorta during atherosclerosis (33). Therefore, Cluster 0 cells were identified as inflammatory macrophages. Cluster 1 was enriched with tissue-resident macrophage markers *Lyve1*, *F13a1*, and typical M2-like macrophage marker *Cd209d*, *Mrc1* (Figures 2C,E). Interestingly, a recent study on ATM using scRNA-seq technique has identified a subpopulation of vascular-associated ATM (VAM) with highly similar enriched genes (34). In addition to *lyve1*, *cd209d*, and *Mrc1*, Cluster 1 cells share other enriched genes with VAM such as *Abca1*, *Stab1*, *Mertk*, and *Lrp6* (Supplementary Figure S2D). So, Cluster 1 cells were identified as VAM. Cluster 2 was highly enriched with *Ccr2*, commonly found in monocytes, which use it as a receptor for CCL2 and chemoattraction. They also showed another monocyte marker, *Lyz1,* and cavity macrophage markers *Cd226*, *Ear2*, *Fn1* (Figures 2E and Supplementary Figure S2A), as described before (35). Thus, Cluster 2 cells appeared to be macrophages differentiated from monocytes attracted from circulation or peritoneal cavities. We decided to follow the nomenclature assigned to both aorta and adipose tissue macrophages (35, 36) and address Cluster 2 cells as cavity macrophages.

Cluster 3 was enriched with a transcriptional signature highly overlapping with previously identified lipid-associated macrophages (LAM) from obese adipose tissues (11). Firstly, these cells are characterized by the enrichment of *Trem2* (Figures 2E,F), a lipid-binding cell surface receptor (37). Secondly, they shared many enrichment genes like *Fabp5*, *Gpnmb*, *Cd9*, *Lgals3*, *Ctsl*, *Ctsd*, *Atp6v0d2*, *Lipa*, and *Lpl* (Figures 2C–F) (11). Interestingly, most of the Trem2^+^ LAM-enriched genes are also enriched in aorta-associated foam cells (33, 35), probably due to the notion that both of them are highly active in lipid metabolism. Cluster 5 cells were enriched with cell proliferation and cell cycle marker genes *Mki6* (38), *Stmn* (39), and *Top2a* (40) (Figures 2C,E). Cluster 6 expressed the highest levels of MCM genes (*Mcm2, Mcm3, Mcm5, Mcm6*) (Figures 2C,E), which encode proteins mediating the DNA replication (41), the S phase of the cell cycle. Therefore, both Cluster 5 and Cluster 6 cells showed signs of proliferation and were identified as proliferating macrophages.

### The pgWAT ATM are highly dynamic during atherogenesis

**Figure 3:**
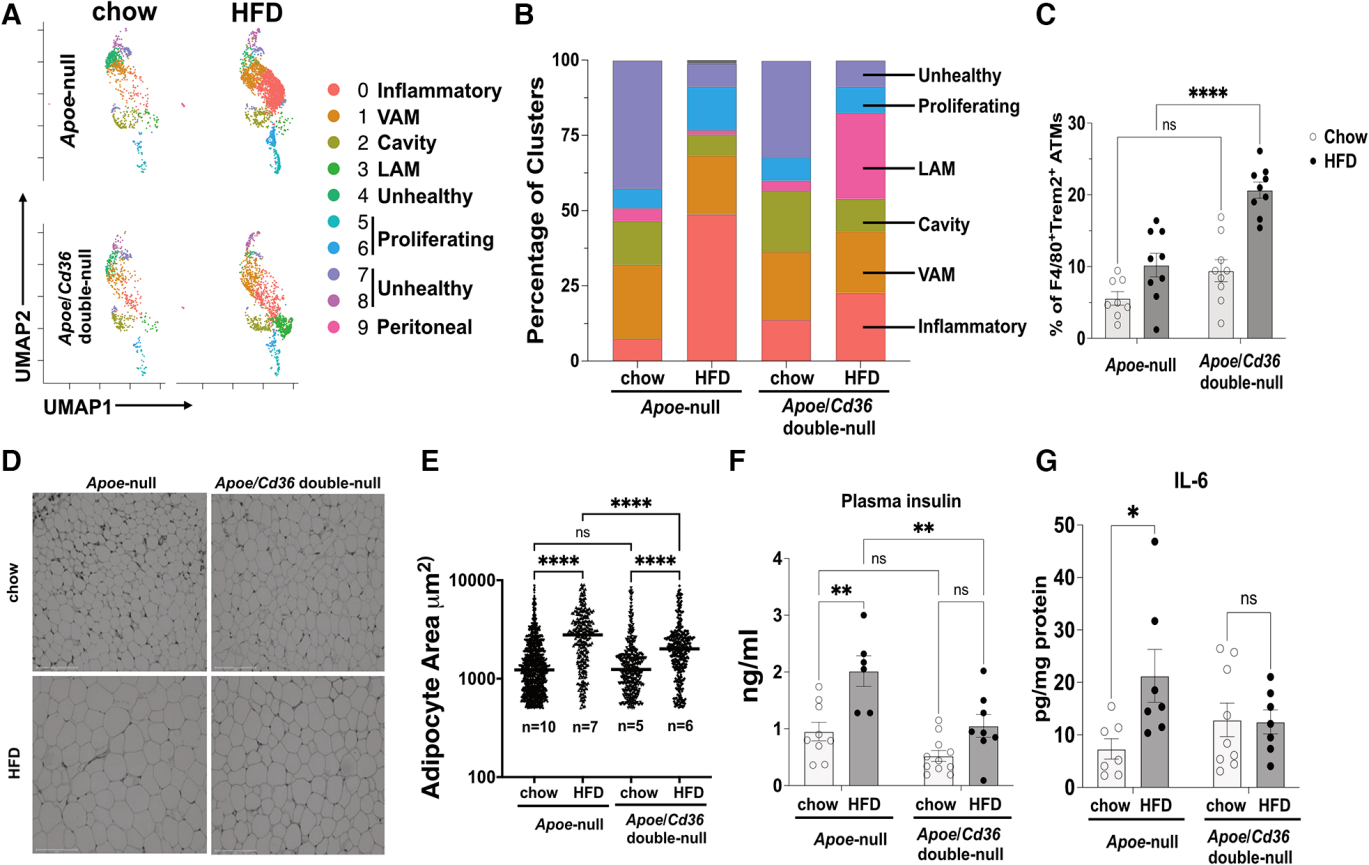
LAM relative content is upregulated by HFD specifically in the *apoe*/*Cd36* double-null mice. (**A**) UMAP plots of 4 different conditions demonstrate distinct ATM constitutions. (**B**) Bar chart of the relative frequency of ATM subpopulations among 4 conditions. (**C**) Flow cytometry analysis of the percentage of F4/80 ^+ ^Trem2^+^ ATM among all CD11b ^+ ^F4/80^+^ ATM. Data are shown as mean ± S.E. in the bar graphs (*n* = 8-9 individuals per group) and Two-Way ANOVA was performed. (**D**) Representative images of H&E-stained adipose tissues. Scale bar: 150 μm. (**E**) Areas of the adipocytes shown in (**D**) were quantified and shown in the dot plot chart. The medium value in each condition was denoted with a horizontal line. The *n* value means the number of individuals. One-Way ANOVA was performed. ns: not significant. (**F**) Plasma insulin levels and (**G**) pgWAT IL-6 levels were shown in the bar graphs (*n* = 6-11 individuals per group). *P < 0.05, **P < 0.01, ****P < 0.0001.

Visceral adipose tissue inflammation promotes atherosclerosis (42). As expected, feeding *Apoe*-null mice with atherogenic HFD significantly increased body weight compared to those on the chow diet (Supplementary Figure S3A). This effect was attenuated in *Apoe*/*Cd36* double-null mice, although no significant difference in subcutaneous or gonadal fat (pgWAT) was observed between *Apoe*-null and *Apoe*/*Cd36* double-null on HFD (Supplementary Figure S3B). Next, we examined the dynamics of pgWAT ATM subpopulations. Figure 3A shows the difference in macrophage subtype distribution. Specifically, inflammatory macrophages increased from 7.4% to 48.8% by HFD in *Apoe*-null pgWAT, while the increase by HFD was mild (13.7% in chow compared to 22.6% in HFD) in *Apoe*/*Cd36* double-null pgWAT (Figure 3B). This is consistent with the notion that atherosclerosis is a systemic inflammatory condition and CD36 contributes to adipose tissue inflammation (19). Nevertheless, VAM did not show obvious change, while cavity macrophages were slightly reduced in both *Apoe*-null pgWAT (14.5% in chow compared to 7.0% in HFD) and *Apoe*/*Cd36* double-null pgWAT (20.3% in chow compared to 10.8% in HFD), probably because more cavity macrophages were differentiated into inflammatory macrophages by HFD. The proliferating macrophages increased from 6.5% to 14.5% by HFD in *Apoe*-null pgWAT. However, a minor increase was observed (7.6% to 8.6% by HFD) in *Apoe*/*Cd36* double-null pgWAT. A surprising finding was the relatively high content of unhealthy macrophages under chow diet conditions (42.5% in *Apoe*-null pgWAT and 32.1% in *Apoe*/*Cd36* double-null pgWAT). Interestingly, they were decreased under HFD conditions (7.6% in *Apoe*-null pgWAT and 8.7% in *Apoe*/*Cd36* double-null pgWAT) (Figure 3B).

The Trem2^+^ LAM distribution pattern among 4 conditions was interesting. While their content was low under chow diet conditions (4.3% in *Apoe*-null pgWAT and 3.4% in *Apoe*/*Cd36* double-null pgWAT), Trem2^+^ LAM percentage increased more than 8-fold to 28.6% in *Apoe*/*Cd36* double-null on HFD, but not in *Apoe*-null on HFD (1.4%) (Figure 3B). To validate, co-immunostaining of F4/80 (macrophage marker) and Trem2, followed by flow cytometry experiments (Figure 3C) confirmed the relatively high amount of Trem2^+^ LAM only in the pgWAT of *Apoe*/*Cd36* double-null mice on HFD. According to a previous report (11), Trem2^+^ LAM counteract inflammation and protect adipose tissue from metabolic dysregulation and adipocyte hypertrophy. Therefore, an increase of Trem2^+^ LAM in *Apoe*/*Cd36* double-null pgWAT HFD condition may be a contributing factor that limited inflammatory macrophage expansion. Supporting this notion, although HFD induced adipocyte hypertrophy in both genotypes, less adipocyte hypertrophy induction was observed in *Apoe*/*Cd36* double-null pgWAT (Figures 3D,E). In addition, HFD increased plasma insulin (Figure 3F) and eWAT IL-6 levels (Figure 3G) in *Apoe*-null mice, both of which indicated insulin resistance (43). Nevertheless, *Apoe*/*Cd36* double-null mice appeared to be protected here, supporting the notion that Trem2^+^ LAM provide beneficial effects against metabolic dysfunction.

### The LAM were less inflammatory and more active in lipid metabolism

**Figure 4:**
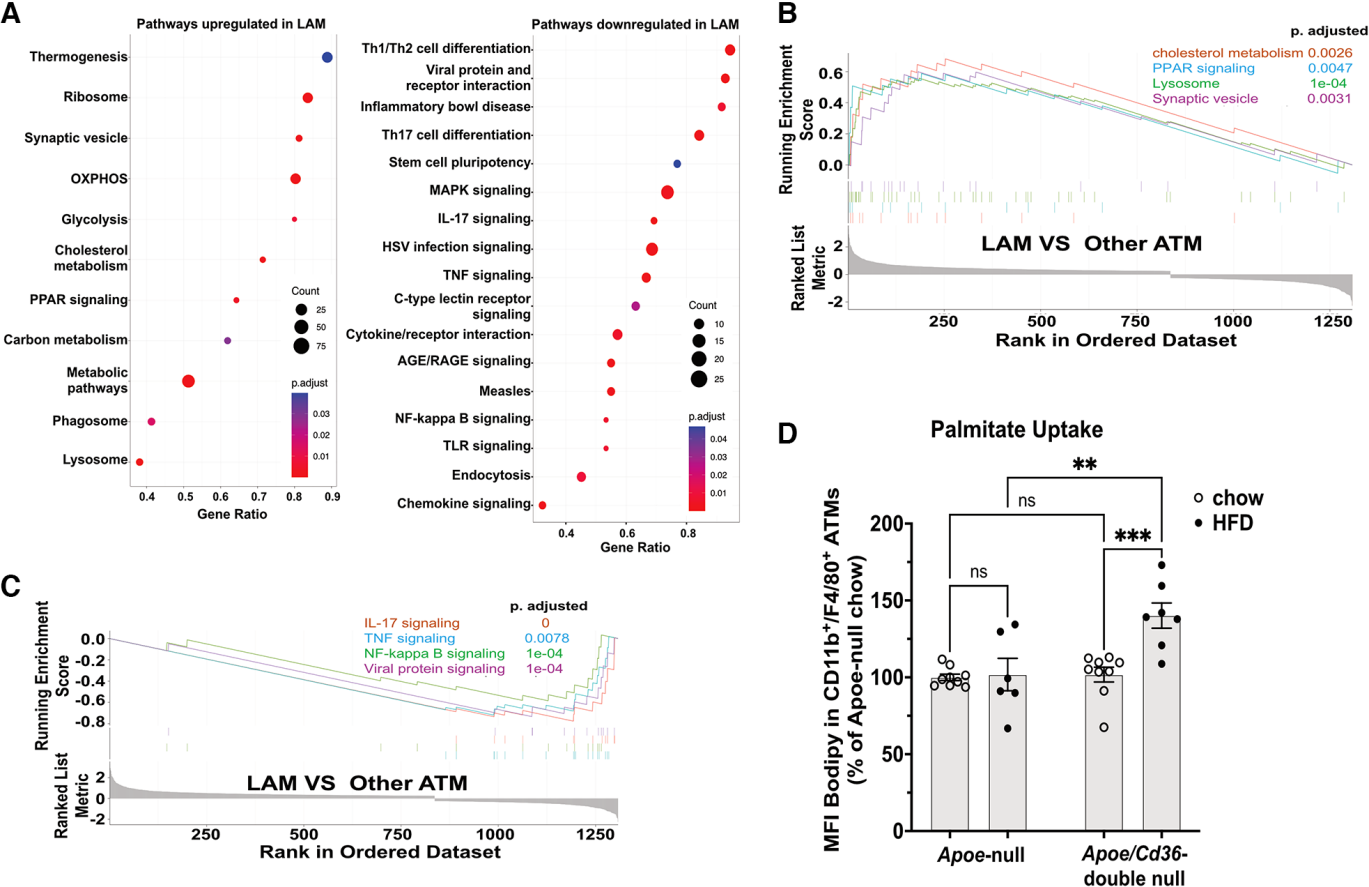
LAM are more active in lipid metabolism and less inflammatory, compared to other ATM. (**A**) Gene ontology enrichment analysis of biological pathways showing upregulated pathways (left panel) and downregulated pathways (right panel) in the LAM. (**B**) GSEA on LAM upregulated pathways including cholesterol metabolism, lysosome, PPAR signaling, and synaptic vesicle cycle. (**C**) GSEA on LAM downregulated pathways including IL-17 signaling, NF-κB signaling, TNF signaling, and viral protein signaling. (**D**) Isolated ATM were incubated with Bodipy-labeled palmitate for 15 min. Flow cytometry analysis of palmitate uptake by CD11b ^+ ^F4/80^+^ ATM. Mean fluorescence intensity (MFI) of Bodipy was quantified. Data are shown as mean ± S.E. in the bar graphs (*n* = 7 individuals per group) and Two-Way ANOVA was performed. ns: not significant. **P < 0.01.

We next investigated pathways that are distinctly activated in major macrophage subpopulations (cluster 0–4). We ranked all genes by their relative enrichment in different macrophage clusters, followed by Gene Set Enrichment Analysis (GSEA) comparing either of two clusters according to Kyoto Encyclopedia of Genes and Genomes database (KEGG). We found that there were very few (2–7) differentially activated/suppressed pathways from a total list of 341 pathways among inflammatory, VAM, cavity, and unhealthy macrophages (Supplementary Table 2). However, the LAM displayed more (35–60) differentially activated/suppressed pathways compared with either major macrophage subpopulations (Supplementary Tables 3-6), suggesting that LAM cells are highly distinct from other ATM. To further understand the features of LAM cells, we combined major ATM other than LAM (denoted as other ATM) and performed a GSEA comparing with LAM. This approach reveals the common pathways that are specifically up or downregulated in LAM (Figure 4A). Consistent with a previous report on the LAM isolated from mouse adipose tissues (11), the LAM appeared to be metabolically active in energy production (oxidative phosphorylation and glycolysis), lipid metabolism (cholesterol metabolism and PPAR signaling for fatty acid metabolism), and phagocytosis (phagosome, synaptic vesicle trafficking, and lysosome) (Figures 4A,B). The downregulated pathways were mostly related to inflammation, such as TNF signaling, NF-κB signaling, IL-17 signaling, and viral protein receptor interaction (Figures 4A,C). Interestingly, the high lipid metabolism with low inflammatory signaling signatures in Trem2^+^ LAM were very similar to those observed in aorta-associated Trem2^+^ foam cells (44). To further test the functional relevance, we isolated pgWAT ATM and conducted a palmitate uptake assay. Consistent with an increase in LAM, ATM from the *Apoe*/*Cd36* double-null HFD condition showed a significant 42% increase in palmitate uptake (Figure 4D).

### ATM subpopulations display distinct patterns of transcription factor regulons

**Figure 5:**
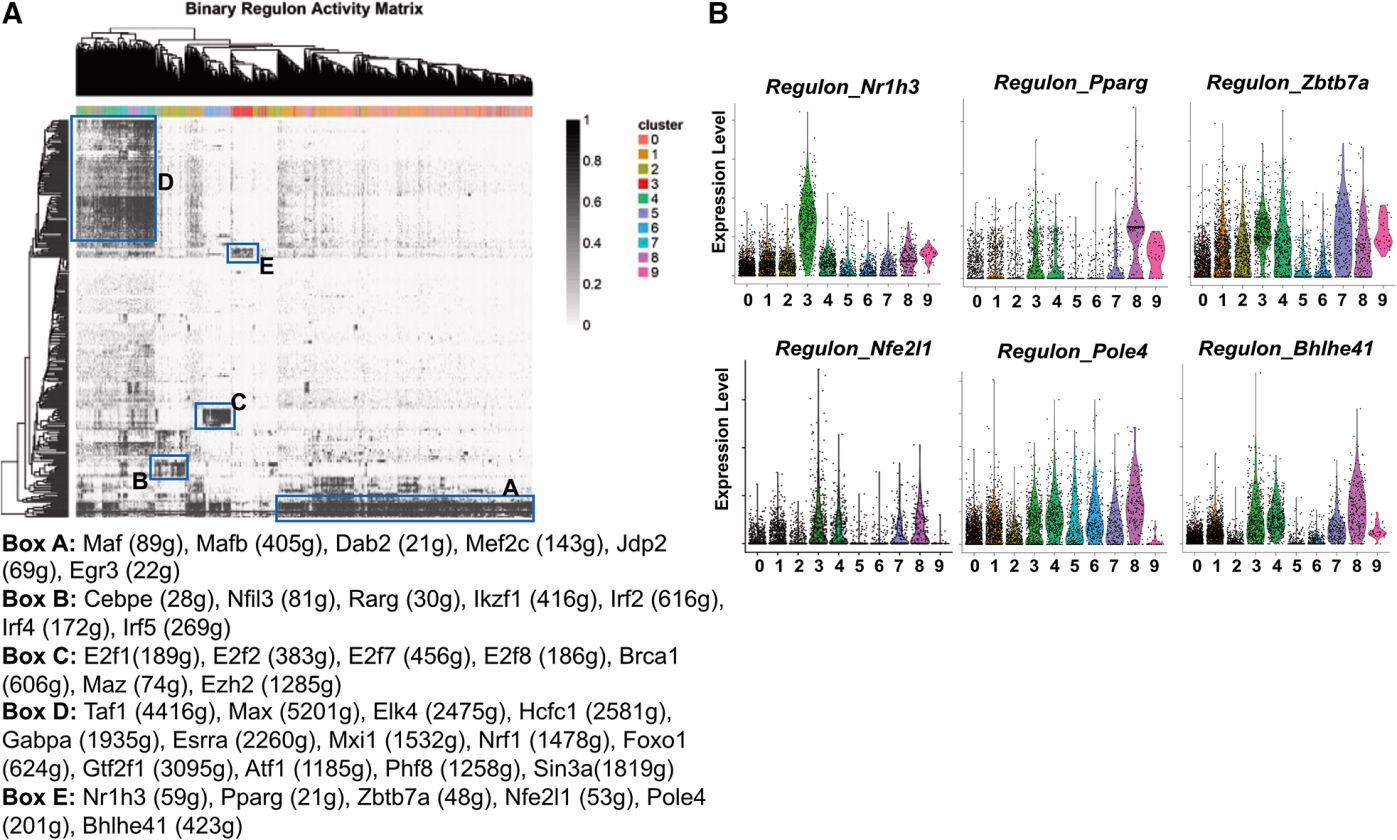
Transcription factor regulons of pgWAT ATM. (**A**) SCENIC results showing the binary regulon activity matrix. The regulons that are specific to ATM subpopulations are highlighted in rectangle boxes (A–E). The highly enriched transcription factor regulons in each box are listed underneath the matrix. (**B**) Violin plots of relative expression of LAM-active regulons among ATM clusters.

Gene transcription is mainly determined by the activities of transcription factors and their co-activators/repressors. To understand what transcription factors, co-activators, co-repressors, and their downstream target genes (regulons) are characteristic of each macrophage cluster, we conducted Single-Cell rEgulatory Network Inference and Clustering (SCENIC) (22). In Figure 5A, we present a comprehensive picture of the regulon activity among all ATM. The first interesting finding is that inflammatory macrophages (cluster 0) and the anti-inflammatory VAM (cluster 1) are similar in regulon activities. Specifically, they share many transcription factor regulons (Box A), including *Maf*, *Mafb*, and *Dab2* that are in favor of “M2-like” anti-inflammatory macrophages (45–47), *Mef2c* that promotes “M1-like” pro-inflammatory macrophages (48), *Jdp2* that is involved in macrophage differentiation (49), and *Egr3*, a regulator of immune cell activation (50).

The cavity macrophages (cluster 2) displayed a distinct pattern of transcription factor regulons (Box B). They include *Cebpe*, which is involved in macrophage lipid metabolisms and differentiation (51, 52), *Nfil3,* which regulates macrophage activation against enteric microbiota (53), and *Rarg, Ikzf1, Irf2, Irf4, Irf5*, all of which facilitate “M2-like” polarization of macrophages (54–56). The proliferating macrophages (clusters 5 and 6) were enriched with transcription factor regulons (Box C). Consistent with their identity, their specific regulons include E2f family members that are critical regulators of the cell cycle (57), *Brca1,* which is an important regulator of DNA damage repair and cell cycle checkpoint control (58), and two transcriptional regulators potentially for genome stability (*Maz, Ezh2*) (59, 60). The unhealthy macrophages (clusters 4, 7, and 8) shared many regulons (Box D), among which are *Nrf1*, a mitochondria biogenesis regulator (61); *Foxo1*, a glycolysis regulator (62), and *Atf1*, which is involved in the macrophage response to iron handling and oxidative stress (63). These findings further suggest that those cells are unhealthy due to defective metabolism, energy production, and subsequent oxidative stress, potentially leading to ferroptosis.

The most interesting finding is the identification of transcription factor regulons that are highly active in LAM (Box E). Firstly, the regulon by *Nr1h3* is almost exclusively highly activated in LAM (Figure 5B). *Nr1h3* encodes the transcription factor Liver X receptor-α (LXR-α), which is a member of the nuclear hormone receptor superfamily. LXR-α is highly activated in tissues/cells for the regulation of cholesterol efflux in response to elevated intracellular cholesterol (64, 65). In macrophages, their activation often leads to anti-inflammatory responses (65), which is in agreement with our pathway analysis showing that LAM are largely non-inflammatory macrophages (Figure 3). LAM is also active in the PPAR-γ (encoded by the gene *Pparg*) regulon (Figure 5B) that is known to regulate macrophage cholesterol levels by mediating CD36 expression and oxidized LDL uptake (66). Additionally, PPAR-γ also controls fatty acid metabolism as well as macrophage polarization in response to different stimulants (67). *Zbtb7a* is a third identified LAM-specific transcription factor (Figure 5B) that is known to promote lipid metabolism through SREBP1 (68). The above three transcription factor regulons (LXR-α, PPAR-γ, and Zbtb7a) have further supported the notion that LAM are highly active in lipid metabolism. The next identified regulon is mediated by the nuclear factor erythroid-2 like 1 (encoded by the gene *Nfe2l1*). It is a member of the CNC-bZIP family of transcription factors, which is induced by oxidative stress (69). Previous studies have indicated that Nrf1 is critical for protecting cells against lipid or metal-induced oxidative stress (70–73). Thus, an active *Nfe2l1*-mediated regulon shared by both LAM and unhealthy macrophages (Figure 5B) suggests that LAM are possibly under oxidative stress due to lipid or metal accumulation. A further demonstration of LAM under stress is the concentration of *Pole4*-mediated regulon. *Pole4* encodes the DNA polymerase *ε* 4, which was found to be a histone chaperone for the maintenance of chromatin integrity (74, 75). Since oxidative stress may lead to DNA damage and cell death (76), the *Pole4* regulon shared by all ATM (Figure 5B) indicated a microenvironment with oxidative stress. The last identified LAM-active regulon is *Bhlhe41*, which is involved in macrophage self-renewal and cell identity (77).

### CD36 deficiency reverses the pro-inflammatory and metabolic responses to HFD

**Figure 6:**
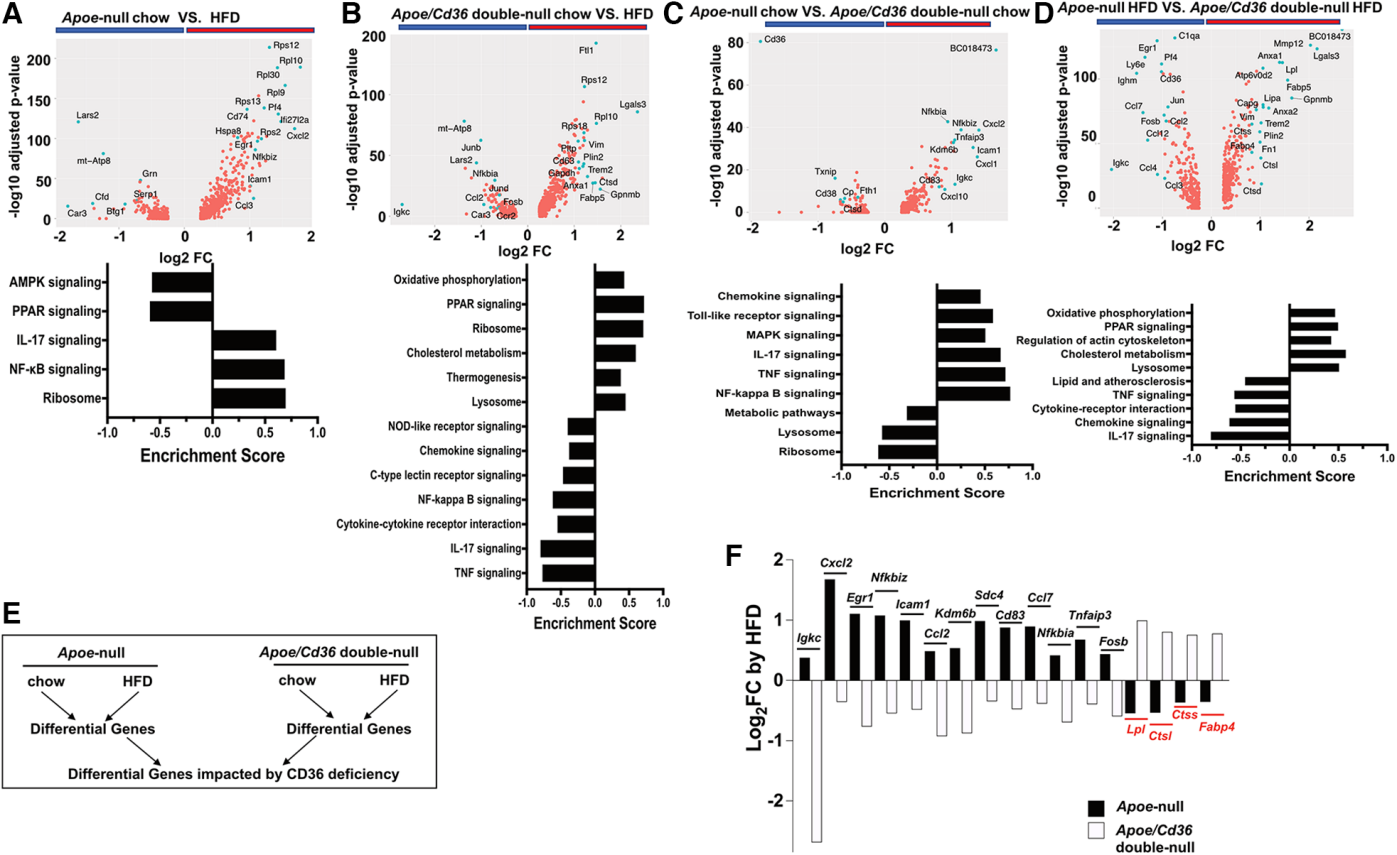
Cd36 deficiency reverses the pro-inflammatory and metabolic responses to HFD. (**A**) Upper panel: Volcano plot showing *Apoe*-null chow vs. *Apoe*-null HFD gene expression fold change (x-axis, log2 scale) and their adjusted *p*-value (y-axis, -log10 scale). Highly significant genes are indicated by a green dot. Bottom panel: GSEA showing significantly different pathways compared between *Apoe*-null chow and *Apoe*-null HFD. (**B**) Same analysis as in (**A**) comparing *Apoe*/*Cd36* double-null chow and *Apoe*/*Cd36* double-null HFD. (**C**) Same analysis as in (**A**) comparing *Apoe*-null chow and *Apoe*/*Cd36* double-null chow. (**D**) Same analysis as in (**A**) comparing *Apoe*-null HFD and *Apoe*/*Cd36* double-null HFD. (**E**) The schematic diagram shows the strategy for identifying genes that are most impacted by CD36 deficiency. (**F**) Fold change (y-axis, Log2 scale) of individual genes (x-axis) as induced by HFD in *Apoe*-null or *Apoe*/*Cd36* double-null pgWAT ATM respectively. The top 17 differentially regulated genes are shown.

Using volcano plots, we showed genes that were differentially expressed by comparing *Apoe*-null chow and *Apoe*-null HFD conditions (Figure 6A). We found many ribosome genes (*Rps12, Rpl10, Rpl30, Rpl9, Rps13, Rps2*) and *Hspa8* for proper protein folding in HFD conditions, indicating ATM were active in protein synthesis. Meanwhile, many inflammatory genes (*Ifi27l2a, Cd74, Cxcl2, Nfkbiz, Icam1, Ccl3*) were significantly elevated, which was confirmed by GSEA (Figure 6A), consistent with the notion that HFD stimulated a pro-inflammatory response in pgWAT.

However, the response of *Apoe*/*Cd36* double-null pgWAT ATM to HFD was largely different. HFD condition was associated with many metabolic genes (*Trem2, Plin2, Ftl1, Pltp, Anxa1, Lgals3, Gapdh*), most of which were involved in lipid metabolism. Other upregulated genes by HFD included *Vim* (cytoskeleton regulation), *Ctsd* (lysosome functions), *Cd63* (exosome biogenesis), and ribosome genes (Figure 6B). Opposite to what was observed in *Apoe*-null pgWAT ATM, inflammatory genes (*Igkc, Junb, Jund, Fosb, Nfkbia, Ccl2, Ccr2*) were downregulated by HFD in *Apoe*/*Cd36* double-null pgWAT ATM. Moreover, GSEA confirmed that HFD increased metabolic pathways (oxidative phosphorylation, PPAR signaling, cholesterol metabolism, thermogenesis, lysosome) and decreased pro-inflammatory pathways (Figure 6B). Since most upregulated genes and pathways were the same to those in LAM (Figure 2F, 4A), we reasoned that the major difference was contributed by a percentage increase in LAM (Figure 3B).

We next directly compared between *Apoe*-null and *Apoe*/*Cd36* double-null. Under chow conditions, the most downregulated gene was *cd36* as expected (Figure 6C). But the most upregulated gene, BC018473, encodes a long non-coding RNA with an unknown function, which might be a good candidate for future investigation. Interestingly, CD36 deficiency led to higher inflammatory genes and pathways and lower metabolic pathways (Figure 6C). Nevertheless, under HFD conditions CD36 deficiency promoted metabolic pathways with reduced inflammation (Figure 6D). To further characterize how CD36 deficiency led to differential gene expression, we quantified the fold change of each gene by HFD within each genotype, followed by a comparison between genotypes (Figure 6E). This strategy allowed us to find the most differentially regulated genes considering both HFD and CD36 deficiency factors. Figure 6F showed the highest 17 differentially regulated genes. Consistently, inflammatory genes (*Igkc, Cxcl2, Egr1, Nfkbiz, Icam1, Ccl2, Kdm6b, Cd83, Ccl7, Nfkbia, Tnfaip3, Fosb*) predominated those upregulated by HFD in *Apoe*-null ATM but downregulated by HFD in *Apoe*/*Cd36* double-null ATM. The only non-inflammatory gene was *Sdc4*, which encoded syndecan-4 protein, a heparan sulfate proteoglycan involved in exosome biogenesis (78). 4 genes were downregulated by HFD in *Apoe*-null ATM but upregulated in *Apoe*/*Cd36* double-null ATM. Two (*Lpl, Fabp4*) were lipid metabolism genes, and two (*Ctsl*, *Ctss*) were lysosome enzyme genes.

## Discussion

In this study, we revealed and characterized distinct transcriptomics signatures of major pgWAT ATM subpopulations from a diet-induced atherosclerosis mouse model. Consistent with the literature applying scRNA-seq on adipose tissues and aortas (11, 34), the major ATM subpopulations were identified as inflammatory macrophages, tissue-resident VAM, cavity macrophages, LAM, and proliferating macrophages (Figure 2). In addition, we showed a previously undefined ATM cluster and labeled them as “unhealthy macrophages” based on their low feature and total RNA counts (Supplementary Figure S2B). However, we do not think these “unhealthy macrophages” were simply byproducts during tissue processing that randomly disrupted cell membranes. This is because: (1) although relatively low compared to other ATM, they still expressed between 500 and 2,000 feature counts with comparable macrophage marker gene expression like *Mrc1* (Figure 2B); (2) their percentages among ATM were much higher under chow conditions than those under HFD conditions (Figure 3B), which suggested that their formation was affected by diet-associated microenvironment instead of random tissue processing, and 3. most importantly, our differential gene expression analysis comparing them to the healthy ATM suggested that “unhealthy macrophages” might be undergoing programmed cell death due to abnormal lipid and iron metabolism (Supplementary Figure S2C). Since the adipose tissues provide a lipid-enriched microenvironment (79, 80), these features of “unhealthy macrophages” have raised the question of whether ATM are constantly under stress by extracellular lipids and the atherogenic hyperlipidemia conditions may aggravate the stress, which drives chronic inflammation in the adipose tissues. Supporting this notion, a previous study in obese mice demonstrated that adipocyte-derived lipids modulated ATM lipotoxicity and M1 (pro-inflammatory) polarization (81). Although we are still unclear about the molecular mechanism of adipocyte/macrophage crosstalk to maintain the local lipid balance under physiological and atherogenic conditions, our findings on LAM elevation in CD36 deficient mice may provide a clue.

LAM is characterized by the enrichment of *Trem2*, a lipid receptor and regulator (82). First identified in WAT from obese mice and humans, the Trem2^+^ LAM function in lipid uptake and metabolism to prevent adipocyte hypertrophy, local inflammation, and systemic metabolic dysregulation (11). The transcriptomics signatures of Trem2^+^ LAM reported here and by Jaitin et al. are highly similar to the Trem2^+^ foam macrophages identified in the mouse aortas (33, 44), which suggests that they may perform similar functions as well. However, a critical difference in our findings is that Trem2^+^ LAM from pgWAT did not appear to accumulate under atherogenic conditions (Figure 3B). Surprisingly, although *Cd36* is one of the markers of Trem2^+^ LAM (11), its deficiency leads to the increase of LAM induced by HFD (Figure 3B). Thus, we draw two conclusions based on these results. First, CD36 is not required for LAM formation and function. Second, CD36 expression restricts pgWAT LAM increase under atherogenic conditions. The increase in Trem2^+^ LAM in *Apoe*/*Cd36* double-null pgWAT was accompanied by less inflammatory macrophages compared to *Apoe*-null pgWAT-HFD. We propose that increased Trem2^+^ LAM alleviated extracellular lipid stress in the adipose tissues and reduced local inflammation. Our pathway analysis (Figure 4) and transcription factor regulon (Figure 5, Box E) result further support this notion showing the enrichment of the lipid metabolism genes and downregulation of the pro-inflammatory genes in Trem2^+^ LAM, which is corroborated by palmitate uptake assay (Figure 4D). In agreement with this hypothesis, we reported that ATM isolated from HFD-fed CD36 deficiency mice were protected from diet-induced pro-inflammatory signaling and insulin resistance (19). Since insulin resistance is associated with hyperinsulinemia (83), it is also consistent with our observation of no plasma insulin induction by HFD in the *Apoe*/*Cd36* double-null mice (Figure 3F).

A potential scenario to explain the increase in Trem2^+^ LAM and less inflammatory macrophages in CD36 deficient mice is that ATM CD36 expression is important for a pro-inflammatory response to hyperlipidemia but is dispensable for lipid uptake and catabolism. Alternatively, as we used CD36 full-body deficient animals, it is also possible that adipocyte CD36 mediates the pro-inflammatory response to hyperlipidemia and restricts Trem2^+^ LAM via adipocyte/macrophage crosstalk. Thus, more CD36 tissue-specific deficient animals are required to study mechanisms of ATM dynamics during atherogenesis further. Regardless of the real scenario, an important lesson learned here is that macrophage lipid metabolism is somehow associated with its inflammatory status and viability. This may also apply to the aortic macrophages that directly contribute to the atherosclerotic plaques. Moreover, the adipose tissues are active endocrine organs and regulate systemic inflammation. Perivascular adipose tissues mediate inflammation that contributes to atherosclerosis (84), which is also compatible with the proposition that adipocytes chronically activate aortic macrophages facilitating the progression of atherosclerosis. Unfortunately, little is known about the molecular mechanisms of how adipocytes crosstalk with macrophages in distinct tissues during atherogenesis.

Our study provides a novel perspective on the pro-atherogenic function of CD36, which is highly expressed by both adipocytes and macrophages. CD36 deficiency under atherogenic conditions not only significantly alters the ATM dynamics (Figure 3B), but also decreases adipocyte hypertrophy (Figures 3E,F), which is associated with adipose tissue dysfunction and metabolic disease (85). Therefore, it is likely that CD36 is involved in the adipocyte/macrophage crosstalk by sensing the extracellular lipids such as oxPC_CD36_ or oxidized LDL, which are highly induced within the circulation under atherogenic hyperlipidemic oxidative conditions (86, 87). These lipids may chronically bind and activate CD36 in both adipocytes and macrophages, leading to adipose tissue dysfunction and abnormal lipid metabolism and deposition, as observed in atherosclerosis. Since CD36 null humans are common in some areas and they appear to have a normal life expectancy and no obvious increased risk for specific diseases (14), our results highlight the possibility of designing specific CD36 inhibitors against chronic inflammatory metabolic diseases, such as atherosclerosis.

Taken together, our results reveal a delicate balance between lipid metabolism and inflammation that governs ATM dynamics during atherogenesis. CD36 is critically involved in the maintenance of this balance, possibly via its dual role as a signal transducer and a fatty acid transporter (14).

### Limitations of the study

Despite our comprehensive analysis integrating scRNA-seq data with tissue histology, plasma profiling, and metabolic assays, we acknowledge the inherent limitations of our study stemming from methodological constraints. Firstly, the “unhealthy macrophages” remain to be further characterized to confirm that they are unhealthy due to defective metabolism induced by lipid stress. Secondly, we could not compare ATM transcriptomics profiles between sexes, although we did not detect obvious sex differences in tissue histology, plasma profiling, and lipid uptake, except for the fat weight. Thirdly, our study employed CD36 full-body knockout mice. As CD36 is highly expressed in both adipocytes and macrophages, the use of cell-type-specific CD36-deficient murine models would further enhance our understanding of CD36's role in adipose tissue function during atherogenesis.

## Data Availability

The datasets presented in this study can be found in online repositories. The accession number for the raw sequence data and the processed data reported in this paper is GSE242120. The code used for scRNA-seq data analysis is available at: https://github.com/vayac/20201109SingleCell. Any additional information required to reanalyze the data reported in this paper is available from the corresponding upon request.
