# Supplementary material for: CD36 restricts lipid-associated macrophages accumulation in white adipose tissues during atherogenesis
Source: Front Cardiovasc Med. 2024 Aug 2;11:1436865. doi: 10.3389/fcvm.2024.1436865 (PMC11327822; doi:10.3389/fcvm.2024.1436865)
Supplement: Supplementary file 1 [file Datasheet1.pdf]

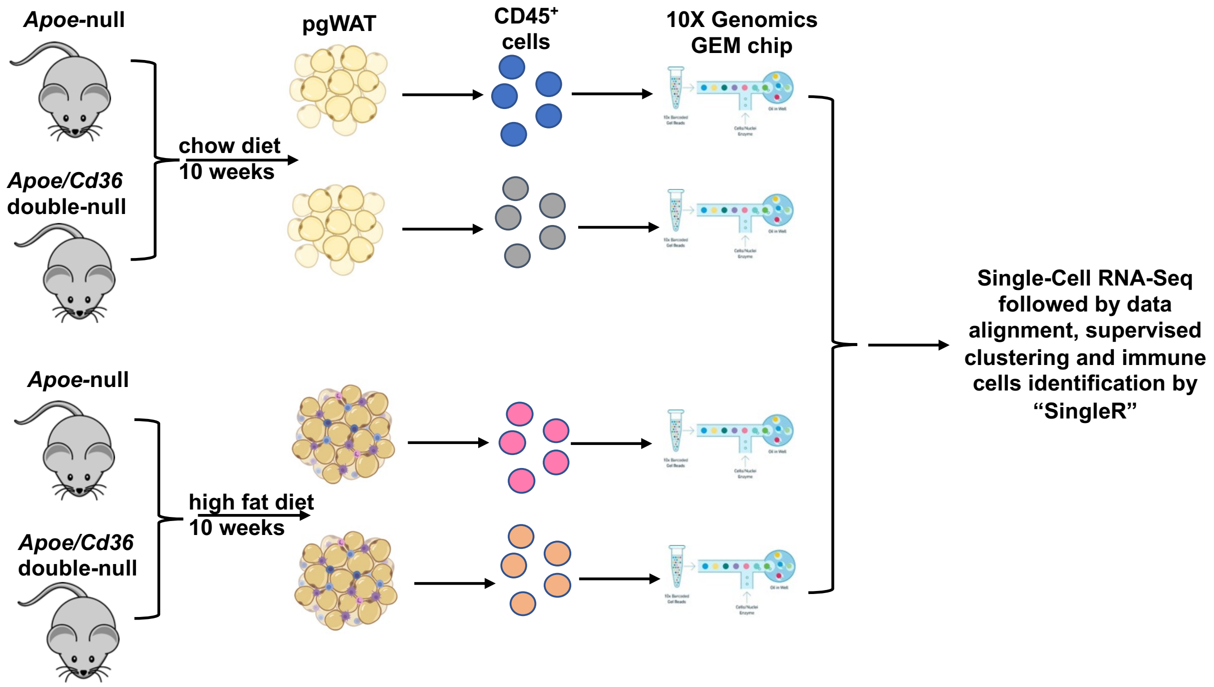

Figure S1. Schematic diagram of the scRNA-seq experimental design. Related to Figure 1.

Fig. S2

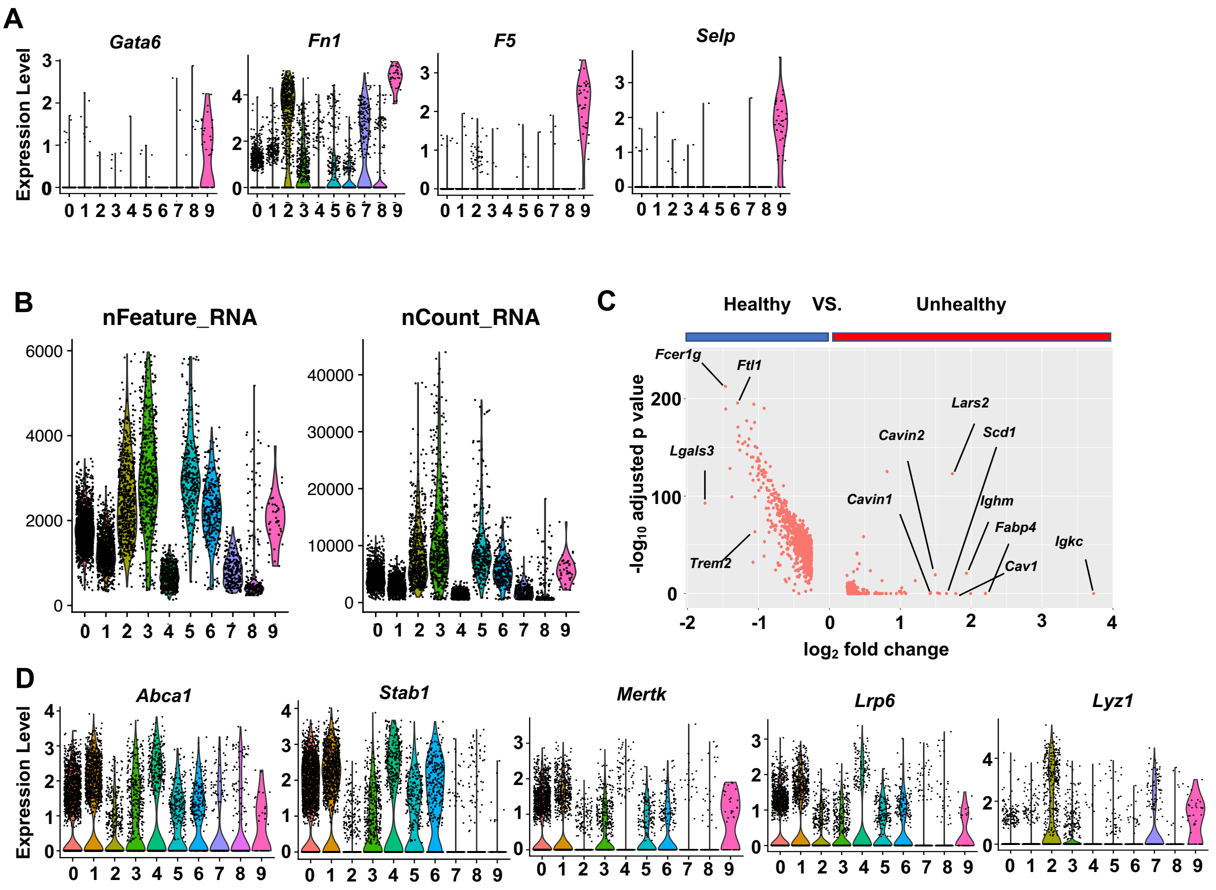

Figure S2. Identification of pgWAT macrophages subpopulations using specific markers. Related to Figure 2. (A) Violin plots of peritoneal macrophage markers. **(B)** The feature RNA counts (left panel) and total RNA counts (right panel) among 10 ATM clusters. **(C)** Volcano plot of genes enriched in healthy vs. unhealthy ATM clusters. **(D)** Violin plots of VAM markers.

Fig. S3

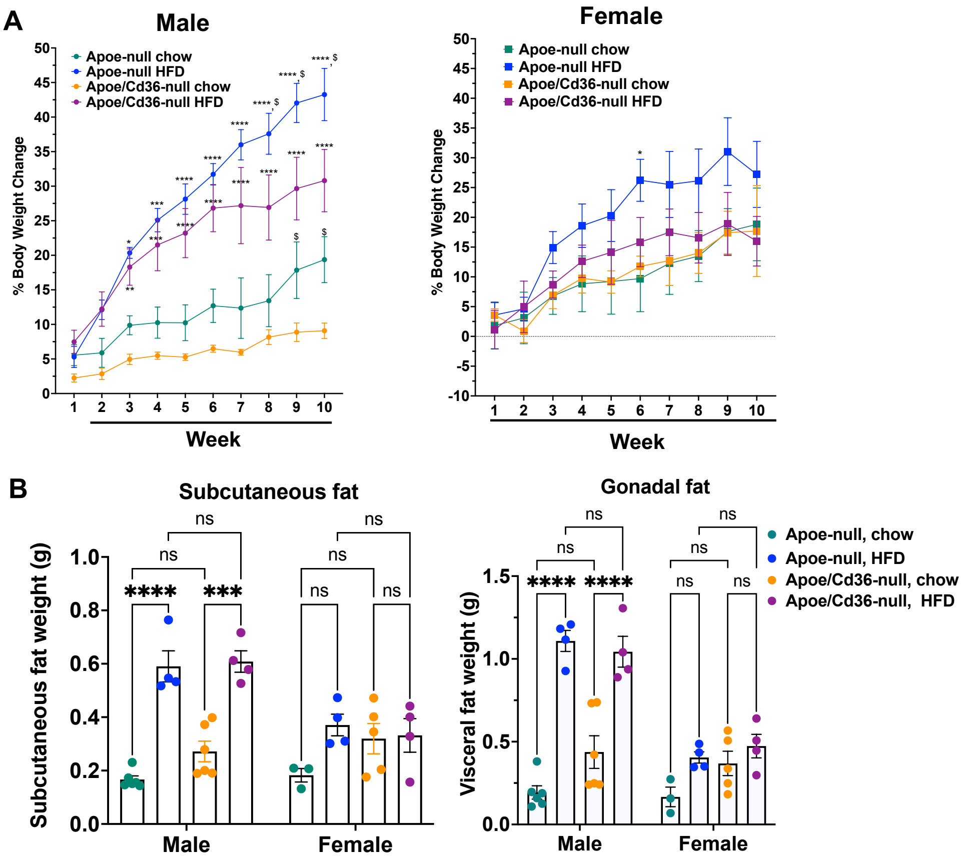

Figure S3. Body weight and fat weight changes introduced by genotype and diet. Related to Figure 3. (A) *Apoe*-null or *Apoe/Cd36* double-null male (left panel) and female (right panel) mice were fed with chow or HFD for 10 weeks. Body weight was measured once a week until week 10. n=10 individuals in each group for both sexes. * indicates significant difference between chow and HFD groups. $ indicates significant difference between genotypes. **(B)** After 10-week diet feeding, all mice were euthanized and subcutaneous fat were weighed. n=4-6 in each group. ns, not significant. ***, p<0.001, ****p<0.0001.

Table S1. Top20 most enriched genes in each ATM. Related to Figure 2.						
p_val	avg_log2FC	pct.1	pct.2	p_val_adj	cluster #	gene
6.8E-291	0.85757856	1	0.966	2.2E-286	0	Cd74
5.7E-270	1.19863298	0.978	0.709	1.8E-265	0	Pf4
1.2E-240	0.85056853	0.995	0.823	4E-236	0	C1qb
2.3E-177	0.69646714	0.995	0.919	7.4E-173	0	H2-Eb1
4.1E-174	0.70607851	0.99	0.786	1.3E-169	0	Fcer1g
5.6E-143	0.76419433	0.929	0.616	1.8E-138	0	Rpl10
6.4E-142	0.80631354	0.786	0.436	2.1E-137	0	Aif1
6.7E-127	0.70708803	0.89	0.622	2.1E-122	0	Cd81
2.7E-121	1.01303567	0.765	0.473	8.6E-117	0	Sdc4
1.7E-120	0.77670192	0.744	0.427	5.5E-116	0	Marcksl1
4.9E-118	0.68889186	0.965	0.793	1.6E-113	0	Egr1
1.9E-110	0.70192349	0.886	0.597	6.2E-106	0	Mgl2
3.4E-109	0.97498416	0.865	0.67	1.1E-104	0	Cxcl2
5.4E-102	0.81254458	0.79	0.55	1.74E-97	0	Cd83
1.1E-83	0.69820372	0.714	0.486	3.54E-79	0	Cited2
6.74E-66	0.76831992	0.453	0.229	2.18E-61	0	Vcam1
5.83E-62	0.77157415	0.359	0.159	1.88E-57	0	Ccl12
2.25E-52	0.89663355	0.357	0.182	7.26E-48	0	Ccl3
8.33E-42	0.94410132	0.371	0.209	2.69E-37	0	Ccl4
1.02E-39	0.69369232	0.421	0.248	3.29E-35	0	Ccl7
4.9E-141	0.96732099	0.937	0.807	1.6E-136	1	F13a1
1.6E-129	0.7582934	0.982	0.902	5E-125	1	Selenop
1.4E-116	0.69106614	0.97	0.905	4.6E-112	1	Mrc1
2.61E-88	0.8116915	0.789	0.599	8.42E-84	1	Gas6
2.7E-81	0.88089116	0.78	0.654	8.71E-77	1	Clec10a
6.92E-74	0.70263164	0.945	0.859	2.23E-69	1	Mafb
2.2E-65	0.69540069	0.867	0.74	7.1E-61	1	Zfp36l1
1.34E-60	0.73091143	0.759	0.593	4.32E-56	1	Stab1
2.38E-60	1.24669039	0.408	0.209	7.69E-56	1	Lyve1
4.55E-58	1.01548916	0.393	0.197	1.47E-53	1	Ednrb
3.41E-53	0.80477685	0.682	0.529	1.1E-48	1	Cfh
3.04E-49	0.71402034	0.686	0.561	9.81E-45	1	Abca1
2.02E-47	0.87908327	0.431	0.25	6.53E-43	1	Cd209f
2.35E-47	0.75254901	0.687	0.535	7.6E-43	1	Cbr2
1.19E-40	1.04327134	0.345	0.192	3.85E-36	1	Cd209d
2.75E-40	0.79795187	0.48	0.324	8.87E-36	1	C4b
4.28E-37	0.69493509	0.501	0.366	1.38E-32	1	Rnase4
3.1E-33	0.69213243	0.275	0.14	1E-28	1	Cd209g
3.67E-33	1.01745165	0.412	0.285	1.19E-28	1	Fcna
4.46E-13	0.87095549	0.339	0.264	1.44E-08	1	Ccl8
0	2.65449921	0.728	0.091	0	2	Ear2
0	1.28399911	0.537	0.044	0	2	Cd226
2.9E-250	1.16970871	0.51	0.058	9.5E-246	2	Rab11fip1
5.7E-250	3.14999829	0.834	0.284	1.8E-245	2	Fn1
1.1E-224	1.80168448	0.72	0.164	3.7E-220	2	Tnip3
7E-209	1.6668766	0.944	0.472	2.3E-204	2	Lsp1
1.8E-202	1.21915834	0.498	0.074	6E-198	2	Bhlhe40
6E-198	1.33123842	0.755	0.2	1.9E-193	2	Cytip
4.4E-197	1.54964246	0.97	0.537	1.4E-192	2	Cd52
1.5E-183	1.56989854	0.924	0.374	5E-179	2	Ccr2
4.2E-176	1.45186395	0.932	0.492	1.4E-171	2	Gm2a
8.2E-164	1.50146463	0.927	0.452	2.7E-159	2	S100a4
5.4E-162	3.23346735	0.51	0.113	1.7E-157	2	Lyz1
3.3E-143	1.43545672	0.973	0.68	1.1E-138	2	Btg1
6.5E-134	1.19201342	0.875	0.413	2.1E-129	2	Tmsb10
2.3E-126	1.1838617	0.956	0.634	7.4E-122	2	S100a6
5.2E-124	1.4023302	0.571	0.166	1.7E-119	2	Malt1
6.2E-109	1.19438369	0.958	0.803	2E-104	2	Ccl6
6.83E-90	1.50585148	0.394	0.104	2.21E-85	2	Il1b
8.61E-62	1.61976453	0.961	0.912	2.78E-57	2	Retnla

0	2.92278021	0.475	0.025	0	3	Gpnmb
0	1.98616789	0.698	0.051	0	3	Atp6v0d2
6.3E-297	1.48712252	0.517	0.042	2E-292	3	Il7r
6.1E-276	2.62296988	0.74	0.137	2E-271	3	Fabp5
3.4E-261	2.06193553	0.866	0.24	1.1E-256	3	Trem2
2.4E-251	3.29069384	0.994	0.567	7.8E-247	3	Lgals3
5.3E-225	2.14195342	0.839	0.239	1.7E-220	3	Anxa1
4.3E-212	1.5810565	0.895	0.302	1.4E-207	3	Pld3
1.3E-197	1.51833741	0.998	0.941	4.2E-193	3	Ftl1
1.2E-188	2.30824054	0.983	0.715	3.9E-184	3	Ctsd
3.1E-182	1.70295996	0.944	0.479	1E-177	3	Lipa
1.8E-174	1.83236373	0.905	0.416	6E-170	3	Cstb
1.7E-168	1.62300239	0.862	0.337	5.4E-164	3	Capg
4.4E-163	1.78451743	0.975	0.676	1.4E-158	3	Plin2
1.9E-160	2.74111365	0.45	0.071	6.1E-156	3	Mmp12
8.4E-151	2.11648061	0.983	0.702	2.7E-146	3	Lpl
1.1E-143	1.68373752	0.94	0.486	3.6E-139	3	Anxa2
9.3E-138	1.46114989	0.984	0.845	3E-133	3	Ctss
7.8E-128	1.95564356	0.882	0.434	2.5E-123	3	Ctsl
1.9E-101	1.46689245	0.948	0.671	6.28E-97	3	Prdx1
6.1E-165	1.47020029	1	1	2E-160	4	Gm42418
1.1E-120	0.98912247	1	0.999	3.4E-116	4	mt-Co1
1.7E-111	1.79498372	0.88	0.84	5.5E-107	4	Lars2
2.39E-41	0.76436885	0.857	0.939	7.73E-37	4	mt-Nd3
6.85E-28	1.21892881	0.633	0.596	2.21E-23	4	Igfbp4
7.38E-28	0.9738153	0.797	0.909	2.38E-23	4	Jund
4.51E-24	0.68812758	0.813	0.836	1.46E-19	4	F13a1
3.23E-23	0.71374454	0.906	0.949	1.04E-18	4	Junb
4.37E-22	0.82702336	0.706	0.807	1.41E-17	4	mt-Nd5
4.44E-22	1.04568768	0.635	0.639	1.43E-17	4	Gas6
5.34E-20	0.98164399	0.646	0.684	1.73E-15	4	Clec10a
3.73E-16	1.03153192	0.782	0.881	1.2E-11	4	Gm26917
2.22E-14	1.22501376	0.396	0.317	7.18E-10	4	mt-Atp8
8.98E-14	0.85829308	0.307	0.204	2.9E-09	4	Car3
3.1E-11	0.73004319	0.661	0.758	1E-06	4	Txnip
1.74E-10	1.08707041	0.363	0.281	5.62E-06	4	Cd209f
2.03E-08	1.52032201	0.39	0.692	0.000655	4	Ighm
9.32E-08	0.9510331	0.254	0.181	0.003008	4	Mmp9
2.75E-05	0.74866596	0.59	0.693	0.889428	4	Cd36
0.004143	0.81379988	0.258	0.237	1	4	Ednrb
0	3.48169059	0.961	0.074	0	5	Top2a
0	3.41627269	0.964	0.077	0	5	Mki67
0	3.26173023	0.984	0.18	0	5	Stmn1
0	2.58173793	0.778	0.017	0	5	Cenpf
0	2.4094025	0.729	0.027	0	5	Ube2c
0	2.22886854	0.82	0.019	0	5	Prc1
0	2.22699289	0.81	0.031	0	5	Pclaf
0	2.2176843	0.856	0.099	0	5	Kif23
0	2.04717326	0.833	0.023	0	5	Birc5
0	1.93949557	0.739	0.032	0	5	Cenpe
0	1.93182019	0.807	0.048	0	5	Racgap1
0	1.927117	0.781	0.014	0	5	Kif11
0	1.91046232	0.889	0.073	0	5	Lmnb1
0	1.87162495	0.637	0.01	0	5	Aspm
0	1.85753246	0.765	0.01	0	5	Nusap1
8.2E-248	2.25745603	0.961	0.219	2.6E-243	5	Tmpo
1.7E-216	2.13511847	0.925	0.232	5.5E-212	5	Smc4
8.8E-214	2.87538685	0.99	0.38	2.8E-209	5	Hmgb2
4E-185	2.75655163	0.997	0.6	1.3E-180	5	Tubb5
2.2E-156	2.093642	0.98	0.616	7.2E-152	5	Tuba1b

2.1E-197	1.18668399	0.498	0.046	6.7E-193	6	Hells
2.9E-179	1.33493694	0.618	0.087	9.5E-175	6	Mcm5
5.7E-178	1.59402121	0.761	0.147	1.8E-173	6	Mcm3
1.1E-174	1.39875945	0.662	0.104	3.5E-170	6	Mcm6
1.5E-166	1.17563075	0.549	0.073	4.9E-162	6	Mcm2
1.3E-152	1.2773272	0.597	0.094	4.2E-148	6	Mcm7
1.5E-130	1.06208527	0.498	0.075	4.9E-126	6	Mcm4
5.6E-125	1.16344345	0.58	0.108	1.8E-120	6	Lig1
8.6E-112	0.96478069	0.392	0.052	2.8E-107	6	Uhrf1
4.8E-99	1.21910345	0.717	0.197	1.54E-94	6	Stmn1
3.3E-82	0.99624775	0.621	0.177	1.07E-77	6	Dnmt1
9.23E-78	0.86664093	0.474	0.11	2.98E-73	6	E2f1
4.34E-72	1.03555413	0.543	0.147	1.4E-67	6	Atad2
1.8E-63	1.25355886	0.846	0.444	5.8E-59	6	Pcna
1.65E-59	1.08733113	0.802	0.362	5.33E-55	6	Ccnd1
3.53E-58	0.88298588	0.461	0.127	1.14E-53	6	Rrm1
2.8E-46	0.84883819	0.853	0.508	9.04E-42	6	Dek
2.54E-45	0.8823399	0.645	0.277	8.2E-41	6	Slbp
2.58E-43	1.03979715	0.925	0.673	8.33E-39	6	Mgl2
2.37E-27	0.86645226	0.488	0.207	7.65E-23	6	Ccl12
1.73E-73	1.55513925	1	1	5.58E-69	7	Gm42418
1.84E-62	1.47893857	0.991	0.992	5.96E-58	7	Malat1
5.21E-49	1.82351175	0.92	0.871	1.68E-44	7	Gm26917
8.09E-27	1.02052763	0.611	0.331	2.61E-22	7	Fn1
9.38E-20	1.16805163	0.783	0.708	3.03E-15	7	Btg1
1.53E-19	1.92212094	0.425	0.215	4.93E-15	7	Tnip3
8.17E-16	1.13707121	0.827	0.86	2.64E-11	7	Neat1
1.58E-14	1.46118827	0.261	0.113	5.09E-10	7	Bhlhe40
9.92E-13	1.38574095	0.496	0.387	3.2E-08	7	Trerf1
1.54E-12	1.24230356	0.553	0.471	4.97E-08	7	Ccdc88a
5.73E-11	1.37428146	0.487	0.394	1.85E-06	7	Fosl2
3.11E-10	1.03982889	0.677	0.7	1.01E-05	7	Slc38a2
2.55E-09	1.48696054	0.323	0.204	8.24E-05	7	Malt1
7.74E-09	1.13397352	0.496	0.392	0.00025	7	Xist
8.09E-09	1.17299137	0.292	0.177	0.000261	7	Sowahc
3.8E-07	1.27776373	0.35	0.255	0.012261	7	Cytip
1.33E-05	1.19168313	0.283	0.205	0.430462	7	Gpr132
6.02E-05	0.99969114	0.403	0.365	1	7	Nabp1
0.000317	1.11501472	0.438	0.435	1	7	Rel
0.002177	1.03165297	0.292	0.256	1	7	Dapk1
2.1E-113	2.86921941	0.46	0.064	6.8E-109	8	Cavin2
3.94E-62	2.93470167	0.384	0.08	1.27E-57	8	Mgll
3.86E-52	2.2619151	0.344	0.074	1.25E-47	8	Cav1
6.93E-49	2.05790899	0.312	0.064	2.24E-44	8	Nedd4
2.51E-47	2.66473384	0.509	0.19	8.11E-43	8	Cavin1
1.16E-46	3.03215081	0.509	0.196	3.74E-42	8	Sparc
4.88E-43	2.17459201	0.263	0.051	1.58E-38	8	Epas1
1.05E-40	1.12323801	1	1	3.38E-36	8	Gm42418
3.61E-37	3.76942332	0.737	0.624	1.17E-32	8	Fabp4
1.02E-28	2.07501985	0.263	0.073	3.31E-24	8	Timp3
2.48E-07	2.09458149	0.451	0.43	0.008003	8	Sptbn1
5.16E-07	2.9650747	0.366	0.293	0.016666	8	Dcn
8.87E-07	1.1230196	0.696	0.849	0.028645	8	Lars2
3.73E-06	0.91868069	0.335	0.608	0.120516	8	Dbi
8.15E-05	2.60820379	0.388	0.679	1	8	Ighm
0.001141	2.17099224	0.254	0.2	1	8	C3
0.005203	0.7455209	0.312	0.489	1	8	Gpx4
0.00759	0.70117824	0.281	0.437	1	8	Klf9
0.007831	0.710677	0.188	0.3	1	8	Nop10
0.008159	1.94468976	0.536	0.662	1	8	Gsn

Comparison between two macrophage clusters. Pathways with significant difference (p.adjust<0.05) are shown.								
Inflammatory vs. VAM								
ID	Description	setSize	enrichmentScore	NES	pvalue	p.adjust	qvalues	rank
mmu03010	Ribosome	81	0.784975211	4.153103	1E-10	2.95E-09	2.42E-09	121
mmu05171	Coronavirus disease - COVID-19	87	0.768489624	4.098877	1E-10	2.95E-09	2.42E-09	121
Inflammtory vs. Cavity								
ID	Description	setSize	enrichmentScore	NES	pvalue	p.adjust	qvalues	rank
mmu04810	Regulation of actin cytoskeleton	45	-0.425692899	-1.957345	0.000439	0.038488	0.03393	335
mmu05142	Chagas disease	20	0.595226564	2.068518	0.000481	0.038488	0.03393	129
Inflammatory vs. Unhealthy								
mmu03010	Ribosome	81	0.776623482	3.337195	1E-10	7.6E-09	6.58E-09	193
mmu05171	Coronavirus disease - COVID-19	100	0.72659566	3.200418	1E-10	7.6E-09	6.58E-09	193
mmu04064	NF-kappa B signaling pathway	16	0.672737429	2.108297	0.000202	0.010226	0.008853	190
mmu05323	Rheumatoid arthritis	24	0.556560952	1.918647	0.001312	0.040917	0.03542	100
mmu04061	Viral protein interaction with cytokine and cytokine recepto	18	0.597986271	1.933815	0.001346	0.040917	0.03542	225
VAM vs. Cavity								
ID	Description	setSize	enrichmentScore	NES	pvalue	p.adjust	qvalues	rank
mmu03010	Ribosome	88	-0.702668545	-3.820621	1E-10	8.85E-09	7.42E-09	278
mmu05171	Coronavirus disease - COVID-19	104	-0.538449328	-3.010648	1E-10	8.85E-09	7.42E-09	281
mmu04714	Thermogenesis	58	-0.427200825	-2.114388	0.000112	0.006605	0.005538	634
mmu04810	Regulation of actin cytoskeleton	38	-0.481332247	-2.117845	0.000374	0.016547	0.013875	280
mmu00190	Oxidative phosphorylation	58	-0.382107468	-1.891203	0.001141	0.040376	0.033856	823
mmu04936	Alcoholic liver disease	17	0.586522058	2.025843	0.001395	0.040395	0.033873	172
mmu04610	Complement and coagulation cascades	14	0.62488992	2.056945	0.001598	0.040395	0.033873	123
VAM vs. Unhealthy								
ID	Description	setSize	enrichmentScore	NES	pvalue	p.adjust	qvalues	rank
mmu03010	Ribosome	60	0.498968471	2.602505	1.08E-07	8.43E-06	7.85E-06	134
mmu05171	Coronavirus disease - COVID-19	67	0.483380957	2.59463	2.77E-07	1.08E-05	1.01E-05	134
mmu04612	Antigen processing and presentation	14	0.614092159	2.135508	0.000467	0.012146	0.01131	128
Cavity vs. Unhealthy								
ID	Description	setSize	enrichmentScore	NES	pvalue	p.adjust	qvalues	rank
mmu03010	Ribosome	89	0.777570766	3.584451	1E-10	1.02E-08	8.58E-09	273
mmu05171	Coronavirus disease - COVID-19	104	0.694953769	3.280923	1E-10	1.02E-08	8.58E-09	273
mmu04514	Cell adhesion molecules	24	0.59686991	2.07892	0.000274	0.018604	0.015647	363

Table S2. Gene Set Enrichment Analysis between either two macrophage subpopulations including inflammatory (cluster 0), VAM (cluster 1), cavity (cluster 2), and unhealthy (cluster 4). Related to Figure 4. Pathways with significant differences (adjusted p-value less than 0.05) are shown.

Inflammatory vs. LAM								
ID	Description	setSize	enrichmentScore	NES	pvalue	p.adjust	qvalues	rank
mmu04657	IL-17 signaling pathway	13	0.844299881	2.739885	1.05E-08	1.9E-06	1.08E-06	62
mmu04668	TNF signaling pathway	24	0.690976665	2.760014	1.41E-07	1.28E-05	7.3E-06	135
mmu01100	Metabolic pathways	189	-0.343262761	-2.098742	3.79E-07	2.29E-05	1.3E-05	302
mmu04061	Viral protein interaction with cytokine and cytokine receptor	18	0.721684292	2.580182	6.89E-07	3.12E-05	1.78E-05	210
mmu05202	Transcriptional misregulation in cancer	32	0.600379082	2.595662	1.04E-06	3.35E-05	1.91E-05	296
mmu05168	Herpes simplex virus 1 infection	38	0.562569192	2.551787	1.11E-06	3.35E-05	1.91E-05	410
mmu04658	Th1 and Th2 cell differentiation	17	0.703185703	2.469189	4.21E-06	0.000109	6.21E-05	399
mmu04659	Th17 cell differentiation	19	0.678349497	2.476848	5.17E-06	0.000115	6.55E-05	399
mmu04142	Lysosome	42	-0.538817181	-2.4131	5.71E-06	0.000115	6.55E-05	260
mmu04966	Collecting duct acid secretion	13	-0.75434099	-2.378581	6.78E-06	0.000123	6.99E-05	287
mmu00190	Oxidative phosphorylation	62	-0.458367624	-2.235302	2.16E-05	0.000355	0.000202	634
mmu05166	Human T-cell leukemia virus 1 infection	44	0.481414867	2.274978	5.14E-05	0.000775	0.000441	410
mmu05150	Staphylococcus aureus infection	16	0.677806548	2.336591	6.29E-05	0.000876	0.000499	162
mmu05417	Lipid and atherosclerosis	46	0.460261153	2.192935	0.000137	0.001766	0.001006	95
mmu05145	Toxoplasmosis	23	0.569109251	2.231122	0.000176	0.0021	0.001197	371
mmu04010	MAPK signaling pathway	42	0.473938336	2.197266	0.000186	0.0021	0.001197	479
mmu04620	Toll-like receptor signaling pathway	17	0.623528404	2.189478	0.000241	0.002346	0.001337	95
mmu04060	Cytokine-cytokine receptor interaction	34	0.505012641	2.198411	0.000256	0.002346	0.001337	210
mmu05162	Measles	23	0.563246234	2.208137	0.000256	0.002346	0.001337	286
mmu05142	Chagas disease	21	0.576635496	2.172723	0.000259	0.002346	0.001337	371
mmu05321	Inflammatory bowel disease	14	0.668849672	2.193707	0.000302	0.00254	0.001448	399
mmu04979	Cholesterol metabolism	14	-0.681946605	-2.250594	0.000309	0.00254	0.001448	331
mmu04062	Chemokine signaling pathway	35	0.491658495	2.168364	0.000388	0.003056	0.001742	223
mmu00010	Glycolysis / Gluconeogenesis	13	-0.669697285	-2.111683	0.000508	0.003617	0.002061	126
mmu05330	Allograft rejection	11	0.691415313	2.115211	0.00052	0.003617	0.002061	410
mmu05332	Graft-versus-host disease	11	0.691415313	2.115211	0.00052	0.003617	0.002061	410
mmu05418	Fluid shear stress and atherosclerosis	27	0.513346562	2.122217	0.000731	0.004903	0.002795	68
mmu04936	Alcoholic liver disease	18	0.580473479	2.075322	0.00098	0.006337	0.003612	371
mmu03320	PPAR signaling pathway	14	-0.64411241	-2.125731	0.001288	0.007775	0.004431	163
mmu04721	Synaptic vesicle cycle	18	-0.592746461	-2.117736	0.001289	0.007775	0.004431	287
mmu05235	PD-L1 expression and PD-1 checkpoint pathway in cancer	17	0.575072208	2.019327	0.001474	0.008385	0.004779	395
mmu04064	NF-kappa B signaling pathway	21	0.528419398	1.991048	0.001482	0.008385	0.004779	196
mmu05320	Autoimmune thyroid disease	10	0.690880989	2.053501	0.00162	0.008885	0.005064	410
mmu05224	Breast cancer	18	0.559739077	2.001192	0.00171	0.009105	0.005189	333
mmu04933	AGE-RAGE signaling pathway in diabetic complications	23	0.51317098	2.011823	0.002042	0.010561	0.006019	47
mmu04940	Type I diabetes mellitus	12	0.624502796	1.96441	0.002674	0.013422	0.00765	410
mmu05207	Chemical carcinogenesis - receptor activation	20	0.518593567	1.90889	0.002744	0.013422	0.00765	223
mmu05144	Malaria	11	0.640418038	1.959197	0.002914	0.01388	0.00791	47
mmu05164	Influenza A	33	0.447706413	1.937383	0.003077	0.014282	0.00814	223
mmu05167	Kaposi sarcoma-associated herpesvirus infection	43	0.39630558	1.853242	0.00347	0.015701	0.008949	410
mmu05133	Pertussis	17	0.546837882	1.920184	0.003671	0.016208	0.009238	139
mmu05140	Leishmaniasis	21	0.496695672	1.871515	0.004021	0.017329	0.009877	144
mmu05163	Human cytomegalovirus infection	45	0.386505946	1.843349	0.004189	0.017632	0.010049	410
mmu01240	Biosynthesis of cofactors	12	-0.64287116	-1.997746	0.004622	0.019011	0.010835	289
mmu05221	Acute myeloid leukemia	11	0.622380533	1.904016	0.005119	0.020592	0.011736	223
mmu05012	Parkinson disease	79	-0.335477032	-1.738136	0.005337	0.020999	0.011968	762
mmu01200	Carbon metabolism	23	-0.49962291	-1.915468	0.006265	0.024128	0.013751	279
mmu05415	Diabetic cardiomyopathy	60	-0.351694143	-1.704136	0.006707	0.025259	0.014396	634
mmu05016	Huntington disease	76	-0.329989342	-1.703187	0.006838	0.025259	0.014396	776
mmu05322	Systemic lupus erythematosus	17	0.524694188	1.842428	0.007155	0.025899	0.014761	181
mmu01230	Biosynthesis of amino acids	15	-0.556270199	-1.858919	0.007418	0.026326	0.015004	279
mmu04621	NOD-like receptor signaling pathway	29	0.433040512	1.816402	0.008177	0.028462	0.016222	196
mmu03010	Ribosome	36	-0.407776628	-1.781264	0.008669	0.029604	0.016873	663
mmu05010	Alzheimer disease	91	-0.304067869	-1.631716	0.009265	0.031054	0.017699	762
mmu05161	Hepatitis B	30	0.42848188	1.817022	0.00967	0.031822	0.018137	475
mmu04714	Thermogenesis	59	-0.34166535	-1.650349	0.010078	0.032574	0.018565	681
mmu04145	Phagosome	48	-0.367081328	-1.700635	0.011507	0.036539	0.020825	223
mmu04928	Parathyroid hormone synthesis, secretion and action	16	0.524583248	1.808387	0.01285	0.040099	0.022854	147
mmu05165	Human papillomavirus infection	46	-0.363274509	-1.65432	0.015035	0.046123	0.026287	201
mmu04917	Prolactin signaling pathway	10	0.60918188	1.810667	0.015433	0.046557	0.026534	223

Table S3. Gene Set Enrichment Analysis between inflammatory macrophage and LAM. Related to Figure 4. Pathways with significant differences (adjusted p-value less than 0.05) are shown.

VAM vs. LAM								
ID	Description	setSize	enrichmentScore	NES	pvalue	p.adjust	qvalues	rank
mmu00190	Oxidative phosphorylation	82	-0.550636707	-2.745401	1E-10	6.63E-09	4.6E-09	578
mmu03010	Ribosome	96	-0.697695008	-3.575834	1E-10	6.63E-09	4.6E-09	319
mmu05171	Coronavirus disease - COVID-19	102	-0.60033128	-3.105394	1E-10	6.63E-09	4.6E-09	319
mmu05012	Parkinson disease	116	-0.424728263	-2.24937	2.79E-07	1.39E-05	9.62E-06	578
mmu04668	TNF signaling pathway	26	0.634916689	2.669696	8.24E-07	3.28E-05	2.27E-05	221
mmu04657	IL-17 signaling pathway	15	0.733178551	2.579143	2.67E-06	7.99E-05	5.54E-05	197
mmu05010	Alzheimer disease	124	-0.397662043	-2.144067	3.2E-06	7.99E-05	5.54E-05	578
mmu04714	Thermogenesis	86	-0.445477763	-2.242491	3.21E-06	7.99E-05	5.54E-05	578
mmu05016	Huntington disease	114	-0.405012473	-2.134195	6.86E-06	0.000152	0.000105	578
mmu04010	MAPK signaling pathway	48	0.474713949	2.341768	8.7E-06	0.000173	0.00012	485
mmu05022	Pathways of neurodegeneration - multiple diseases	131	-0.376725328	-2.045477	1.26E-05	0.000228	0.000158	578
mmu05415	Diabetic cardiomyopathy	92	-0.410684966	-2.08905	1.87E-05	0.000293	0.000203	578
mmu05014	Amyotrophic lateral sclerosis	119	-0.389346062	-2.072631	1.92E-05	0.000293	0.000203	578
mmu01100	Metabolic pathways	238	-0.312240944	-1.845927	2.63E-05	0.000366	0.000254	541
mmu04658	Th1 and Th2 cell differentiation	18	0.653577643	2.417614	2.76E-05	0.000366	0.000254	423
mmu05202	Transcriptional misregulation in cancer	37	0.499861327	2.306947	2.98E-05	0.000371	0.000257	442
mmu04966	Collecting duct acid secretion	14	-0.689605938	-2.14837	9.44E-05	0.001105	0.000766	366
mmu04659	Th17 cell differentiation	20	0.595897354	2.297408	0.000115	0.001273	0.000882	541
mmu04260	Cardiac muscle contraction	25	-0.567364844	-2.111028	0.000145	0.001515	0.00105	465
mmu05208	Chemical carcinogenesis - reactive oxygen species	91	-0.380218042	-1.929402	0.000187	0.001818	0.00126	578
mmu04142	Lysosome	43	-0.494524877	-2.127573	0.000192	0.001818	0.00126	212
mmu05162	Measles	28	0.511994795	2.212042	0.000342	0.002971	0.002059	131
mmu04061	iral protein interaction with cytokine and cytokine recepto	11	0.687342911	2.129525	0.000343	0.002971	0.002059	137
mmu05235	D-L1 expression and PD-1 checkpoint pathway in cance	21	0.556044706	2.17904	0.000385	0.003129	0.002168	698
mmu04928	Parathyroid hormone synthesis, secretion and action	19	0.573684693	2.157314	0.000397	0.003129	0.002168	444
mmu05224	Breast cancer	21	0.55272215	2.16602	0.000409	0.003129	0.002168	367
mmu00010	Glycolysis / Gluconeogenesis	13	-0.670881106	-2.037261	0.000661	0.004869	0.003374	356
mmu05215	Prostate cancer	18	0.582457572	2.154537	0.000765	0.005435	0.003766	643
mmu05020	Prion disease	119	-0.336501378	-1.79132	0.001076	0.007384	0.005116	578
mmu04936	Alcoholic liver disease	18	0.56794071	2.100839	0.001349	0.008951	0.006202	445
mmu05161	Hepatitis B	33	0.430837931	1.926245	0.001839	0.011803	0.008179	652
mmu05168	Herpes simplex virus 1 infection	38	0.413091444	1.913816	0.002112	0.013132	0.0091	546
mmu04550	Signaling pathways regulating pluripotency of stem cells	18	0.535152787	1.979555	0.004033	0.023857	0.016532	643
mmu03320	PPAR signaling pathway	14	-0.581583184	-1.81184	0.004076	0.023857	0.016532	273
mmu04625	C-type lectin receptor signaling pathway	27	0.457019576	1.946206	0.004235	0.024081	0.016686	290
mmu01200	Carbon metabolism	25	-0.48839821	-1.817212	0.004559	0.025203	0.017464	358
mmu05417	Lipid and atherosclerosis	51	0.339163993	1.699496	0.005189	0.027589	0.019118	281
mmu05150	Staphylococcus aureus infection	12	0.590003291	1.881061	0.005318	0.027589	0.019118	257
mmu05321	Inflammatory bowel disease	11	0.602552541	1.866827	0.005438	0.027589	0.019118	546
mmu04620	Toll-like receptor signaling pathway	18	0.524314401	1.939463	0.005546	0.027589	0.019118	652
mmu04144	Endocytosis	50	0.357432055	1.776209	0.006671	0.032377	0.022435	272
mmu01522	Endocrine resistance	22	0.451323844	1.79695	0.007224	0.034227	0.023717	643
mmu04721	Synaptic vesicle cycle	17	-0.552776451	-1.812071	0.008143	0.037687	0.026115	366
mmu04213	Longevity regulating pathway - multiple species	15	0.528015198	1.857428	0.008707	0.03938	0.027288	643
mmu05145	Toxoplasmosis	23	0.435108071	1.772182	0.009602	0.04246	0.029422	652
mmu05207	Chemical carcinogenesis - receptor activation	26	0.43012546	1.80859	0.01078	0.046634	0.032314	662
mmu04932	Non-alcoholic fatty liver disease	73	-0.336136827	-1.642917	0.011245	0.047611	0.032991	578
mmu04610	Complement and coagulation cascades	11	0.572770697	1.774557	0.011596	0.048076	0.033314	257

Table S4. Gene Set Enrichment Analysis between VAM and LAM. Related to Figure 4. Pathways with significant differences (adjusted p-value less than 0.05) are shown.

Cavity vs. LAM								
ID	Description	setSize	enrichmentScore	NES	pvalue	p.adjust	qvalues	rank
mmu04142	Lysosome	54	-0.568735621	-2.736321	3.01E-08	5.09E-06	4.19E-06	233
mmu04514	Cell adhesion molecules	21	0.628717346	2.38419	1.75E-05	0.001069	0.000879	156
mmu03320	PPAR signaling pathway	12	-0.765331758	-2.345559	1.9E-05	0.001069	0.000879	153
mmu04659	Th17 cell differentiation	23	0.590939884	2.340187	2.85E-05	0.001203	0.000989	424
mmu05145	Toxoplasmosis	26	0.578579751	2.332642	4.86E-05	0.001642	0.00135	424
mmu05168	Herpes simplex virus 1 infection	36	0.511514035	2.277348	7.56E-05	0.00213	0.001751	507
mmu05169	Epstein-Barr virus infection	39	0.495875556	2.257599	9.67E-05	0.002334	0.001919	329
mmu04658	Th1 and Th2 cell differentiation	21	0.591436018	2.242813	0.000137	0.002631	0.002163	424
mmu05332	Graft-versus-host disease	10	0.751989084	2.25216	0.00014	0.002631	0.002163	156
mmu04940	Type I diabetes mellitus	11	0.718761202	2.17481	0.000156	0.002637	0.002168	156
mmu04966	Collecting duct acid secretion	14	-0.675994142	-2.142046	0.000177	0.002718	0.002234	345
mmu04064	NF-kappa B signaling pathway	21	0.579884334	2.199008	0.000215	0.003034	0.002495	295
mmu05140	Leishmaniasis	22	0.577838826	2.24198	0.00024	0.003118	0.002563	156
mmu00190	Oxidative phosphorylation	30	-0.515472779	-2.112523	0.000362	0.004225	0.003474	581
mmu04668	TNF signaling pathway	19	0.592432349	2.183207	0.000375	0.004225	0.003474	431
mmu04721	Synaptic vesicle cycle	17	-0.638174793	-2.176401	0.000424	0.004477	0.003681	345
mmu04933	AGE-RAGE signaling pathway in diabetic complications	23	0.516677797	2.046101	0.001021	0.00998	0.008205	348
mmu04625	C-type lectin receptor signaling pathway	20	0.544258715	2.034263	0.001075	0.00998	0.008205	329
mmu04979	Cholesterol metabolism	16	-0.614660622	-2.057864	0.00116	0.00998	0.008205	388
mmu01100	Metabolic pathways	154	-0.298841595	-1.769288	0.001193	0.00998	0.008205	547
mmu05134	Legionellosis	14	0.612374009	2.024295	0.001278	0.00998	0.008205	243
mmu05135	Yersinia infection	21	0.529128641	2.006535	0.001299	0.00998	0.008205	485
mmu05164	Influenza A	31	0.464514672	1.985376	0.001519	0.011165	0.009179	507
mmu05162	Measles	23	0.498396382	1.973705	0.002209	0.015559	0.012792	329
mmu05321	Inflammatory bowel disease	12	0.640689874	2.004936	0.002666	0.01782	0.014651	156
mmu05235	PD-L1 expression and PD-1 checkpoint pathway in cance	13	0.601129963	1.907211	0.002741	0.01782	0.014651	424
mmu05166	Human T-cell leukemia virus 1 infection	33	0.425589959	1.845033	0.003464	0.021683	0.017827	290
mmu05416	Viral myocarditis	16	0.566219854	1.953585	0.003793	0.022896	0.018824	156
mmu04640	Hematopoietic cell lineage	23	0.464930897	1.841178	0.007427	0.043284	0.035587	156

Table S5. Gene Set Enrichment Analysis between cavity macrophages and LAM. Related to Figure 4. Pathways with significant differences (adjusted p-value less than 0.05) are shown.

LAM vs. Unhealthy								
ID	Description	setSize	enrichmentScore	NES	pvalue	p.adjust	qvalues	rank
mmu03010	Ribosome	98	0.742780776	3.323152	1E-10	1.08E-08	8.79E-09	258
mmu05171	Coronavirus disease - COVID-19	110	0.685107932	3.113906	1E-10	1.08E-08	8.79E-09	258
mmu00190	Oxidative phosphorylation	84	0.565074876	2.465377	1.93E-10	1.39E-08	1.13E-08	785
mmu05012	Parkinson disease	133	0.468630646	2.176668	3.77E-08	2.04E-06	1.66E-06	889
mmu05010	Alzheimer disease	140	0.436760072	2.038469	8.99E-07	3.88E-05	3.15E-05	889
mmu05016	Huntington disease	129	0.44580135	2.064084	1.08E-06	3.88E-05	3.15E-05	889
mmu05022	Pathways of neurodegeneration - multiple diseases	151	0.423870333	1.99955	1.49E-06	4.6E-05	3.75E-05	889
mmu05014	Amyotrophic lateral sclerosis	136	0.420749877	1.960667	5.02E-06	0.000136	0.00011	889
mmu05415	Diabetic cardiomyopathy	97	0.450859155	2.011535	9.94E-06	0.000239	0.000194	770
mmu05020	Prion disease	136	0.412640287	1.922877	1.18E-05	0.000255	0.000208	647
mmu04714	Thermogenesis	95	0.459573785	2.053376	1.3E-05	0.000255	0.000208	647
mmu04668	TNF signaling pathway	22	-0.601052492	-2.587931	2.64E-05	0.000475	0.000386	308
mmu04260	Cardiac muscle contraction	25	0.63128568	2.191804	4.02E-05	0.000667	0.000543	684
mmu05208	Chemical carcinogenesis - reactive oxygen species	96	0.433468248	1.935385	5.13E-05	0.000792	0.000645	647
mmu04010	MAPK signaling pathway	52	-0.387665397	-2.181149	0.000127	0.001822	0.001483	482
mmu04657	IL-17 signaling pathway	15	-0.649742392	-2.470989	0.000151	0.001964	0.001598	187
mmu01100	Metabolic pathways	262	0.335742504	1.654072	0.000155	0.001964	0.001598	609
mmu04142	Lysosome	51	0.490624942	1.980034	0.000263	0.003102	0.002524	445
mmu04658	Th1 and Th2 cell differentiation	20	-0.557310192	-2.382235	0.000273	0.003102	0.002524	420
mmu04659	Th17 cell differentiation	22	-0.528562972	-2.275815	0.000509	0.005329	0.004337	399
mmu05202	Transcriptional misregulation in cancer	36	-0.423772182	-2.110033	0.000518	0.005329	0.004337	399
mmu05215	Prostate cancer	19	-0.516018939	-2.1911	0.001781	0.017011	0.013844	597
mmu04550	Signaling pathways regulating pluripotency of stem cells	18	-0.520037709	-2.156703	0.001811	0.017011	0.013844	302
mmu04966	Collecting duct acid secretion	13	0.65095538	1.887371	0.002652	0.023868	0.019425	516
mmu04932	Non-alcoholic fatty liver disease	77	0.399518308	1.726992	0.002923	0.025251	0.02055	770
mmu05168	Herpes simplex virus 1 infection	50	-0.318594007	-1.804437	0.004165	0.033755	0.027471	315
mmu04970	Salivary secretion	12	0.652592189	1.85463	0.004219	0.033755	0.027471	249
mmu05235	PD-L1 expression and PD-1 checkpoint pathway in cance	19	-0.47781011	-2.028859	0.005387	0.041558	0.033821	398
mmu04971	Gastric acid secretion	12	0.63894966	1.815859	0.005833	0.043448	0.03536	249

Table S6. Gene Set Enrichment Analysis between LAM and unhealthy macrophages. Related to Figure 4. Pathways with significant differences (adjusted p-value less than 0.05) are shown.
